# The application of enhanced recovery after surgery (ERAS) in chronic rhinosinusitis patients undergoing endoscopic sinus surgery: A systematic review and meta-analysis

**DOI:** 10.1371/journal.pone.0291835

**Published:** 2023-09-21

**Authors:** Yuqi Wu, Yijie Fu, Yuanqiong He, Xinru Gong, Zhoutong Han, Hongli Fan, Tianmin Zhu, Hui Li

**Affiliations:** 1 School of Rehabilitation and Health Preservation, Chengdu University of Traditional Chinese Medicine, Chengdu, China; 2 School of Preclinical Medicine, Chengdu University, Chengdu, China; Tehran University of Medical Sciences, ISLAMIC REPUBLIC OF IRAN

## Abstract

**Objectives:**

Enhanced recovery after surgery (ERAS) has become extensively practiced and has shown encouraging benefits. Within recent years, ERAS has also been increasingly performed in chronic rhinosinusitis (CRS) patients undergoing endoscopic sinus surgery (ESS). However, the actual efficacy of ERAS in CRS patients undergoing ESS is not completely clear, and the related evidence remains weak. This systematic review and meta-analysis aimed to evaluate the effectiveness and safety of ERAS in the perioperative management of CRS patients receiving ESS.

**Methods:**

We searched randomized controlled trials in PubMed, Web of Science, EMBASE, Cochrane CENTRAL, Ovid, China National Knowledge Infrastructure, Chinese BioMedical Literature Database, Wanfang, and VIP Database up to February 2023, to analyze the effectiveness and safety of ERAS in ESS perioperative management of CRS patients. We appraised the methodological quality in the included RCTs using the Cochrane Collaboration tool and assessed the quality of evidence with the Recommendations Assessment, Development and Evaluation (GRADE) tool. Meta-analysis, subgroup analysis, and sensitivity analysis were carried out with the the software Review Manager 5.3 and Stata 12.0. In addition, potential publication bias was detected by Begg’s test, Egger’s test, and funnel plot test.

**Results:**

Twenty-eight studies involving 2636 patients were included within this study. In comparison with the standard care (SC) group, the ERAS group had the advantages in the following aspects: length of stay (MD = -2.50, 95%CI: -3.04 to -1.97), pain scores (MD = -1.07, 95%CI: -1.46 to -0.67), anxiety score (SMD = -2.13, 95%CI: -2.83 to -1.44), depression score (SMD = -2.42, 95%CI: -3.13 to -1.71), hospitalization expenses, and quality of life. At the same time, the ERAS group presented a markedly lower incidence of adverse events in comparison to the SC group, such as overall complications (RR = 0.28, 95%CI:0.20 to 0.41), postoperative nausea and vomiting (RR = 0.33, 95%CI:0.21 to 0.50), facial edema (RR = 0.20, 95%CI:0.11 to 0.38), low back pain (RR = 0.28, 95%CI:0.16 to 0.49), urinary retention (RR = 0.12, 95%CI:0.05 to 0.30) and haemorrhage (RR = 0.19, 95%CI:0.07 to 0.55).

**Conclusions:**

The results showed that the ERAS protocol is effective and safe in CRS patients who undergo ESS. However, Due to the limited overall methodological quality included studies, caution should be exercised in the interpretation of the results. More high-quality, multiple-centre, and large-sample studies are in demand in the future to further validate its clinical efficacy.

## Introduction

Chronic rhinosinusitis (CRS) is a common chronic disease occurring in people of all ages, often defined as the ongoing inflammation of the nasal cavity and sinuses, with a global incidence of about 5–12% [1]. Depending on whether nasal polyps (NP) are present or not, CRS can be precisely categorized as CRS with NP (CRSwNP) and CRS without NP (CRSsNP) [2]. The main symptoms of CRS are nasal congestion/obstruction, nasal anterior/posterior discharge, loss/diminished sense of smell, or facial pain/pressure that lasts for at least 12 weeks, which significantly has negative impacts on patients’ quality of life (QOL) and even causes psychological problems such as anxiety and depression [2, 3]. CRS patients usually receive medical treatment early in the course of illness, if no remission, surgical treatment will be chosen. Endoscopic sinus surgery (ESS), a delicate and safe operation with the advantages of clear vision, less bleeding and injury, rapid recovery, and great preservation of nasal physiological function [4], has become a prior option for CRS patients with no or inferior response to drug therapy [5]. However, patients who receive ESS might also have a poor prognosis due to a variety of complicating factors [4]. Some patients still reported no improvement in postoperative symptoms and even serious mental illness [3, 6, 7]. It follows that only paying attention to ESS treatment itself may be insufficient. To reduce postoperative problems, both clinicians and patients should attach importance to recovery from surgery. Maybe ESS combined with a systemic surgical care pathway helps to gain a better curative effect.

Enhanced recovery after surgery (ERAS) is an integrated and effective multi-modal, multidisciplinary care program designed to incorporate evidence-based strategies into pre-, intra-, and postoperative care planning to optimize perioperative management, reduce surgical trauma and the body’s inherent responses to promote early recovery after surgery and improve outcomes [8]. ERAS was first innovatively introduced in the mid-1990s by Danish surgeon Henrik Kehlet et al., and was first used in colorectal surgery [8, 9]. After that, ERAS increasingly gained attention in perioperative care and was soon used for other types of surgery. Some surgery departments have designed exclusive ERAS guidelines for certain types of surgery, mainly including the following items such as preoperative education and counseling, fluid management, multimodal analgesia, early feeding and mobilization, etc. Up to now, ERAS management strategies have been applied to a wide variety of surgeries including hepatobiliary [10], gynecological [11], gastric [12], urology [13], vascular [14], and bariatric surgery [15], with good recovery outcomes.

However, it is worth noting that the implementation and development of ERAS protocols in otolaryngology has been relatively slow and there are currently no recommended guidelines for it yet. Although there has been a substantial rise in research regarding the usage of ERAS protocols for CRS patients undergoing ESS over the past 5 years [5, 16], no meta-analysis on this topic has been published to date. More supporting evidence is necessary in clinical practice to validate the efficacy of ERAS protocols in CRS patients receiving ESS. Here, we rigorously carried out a systematic review and meta-analysis to assess, in a combined qualitative and quantitative manner, the clinical effectiveness and safety of the ERAS protocol in CRS patients undergoing ESS compared with standard care (SC). We expect to draw a clear and systematic conclusion from this review and provide Evidence-based support that the ERAS protocols can be safely and effectively applied to CRS patients undergoing ESS, providing a promising rehabilitation scheme for clinicians and patients.

## Materials and methods

### Methods

This meta-analysis was carried out in accordance with the Preferred Reporting Items for Systematic Reviews and Meta-Analyses (PRISMA) guidelines. The protocol was registered in PROSPERO (CRD42022366694).

### Inclusion criteria

#### Participants

CRS patients undergoing ESS;

#### Intervention and comparison

The study group received ERAS care (the adopted ERAS protocol consists of at least six elements), while the control group received conventional SC;

#### Outcomes

Included studies were required to include an outcome of one of the following:

*Primary outcomes included*: *length of stay (LOS)*. Secondary outcomes included:1). complications (including overall postoperative complications, postoperative nausea and vomiting (PONV), facial edema, low back pain, urinary retention, and haemorrhage), 2) pain score (assessed using visual analogue scale [VAS]), 3) QOL (assessed by the 22-item Sinonasal Outcomes Test [SNOT-22]/the 20-Item Sino-Nasal Outcome Test [SNOT-20]/Generic Quality of Life Inventory-74 [GQOL-74]/Or other homemade QOL scales, etc.), 4) anxiety score (assessed by the Self-Rating Anxiety Scale [SAS]/the Hamilton Anxiety Scale [HAMA]/the 7-item Generalized Anxiety Disorder Questionnaire [GAD-7]), 5) depressive score (assessed by the Self-Rating Depression Scale [SDS]/the Hamilton Depression Rating Scale [HAMD]/the Patient Health Questionnaire-9 [PHQ-9]), 6) hospitalization expenses;

#### Study design

Randomized controlled trial (RCT).

### Exclusion criteria

Studies should be excluded if they have the following circumstances: (1) studies that are not RCTs, (2) ERAS protocol contains less than six items, (3) patients received other treatments during the period of ERAS care, (4) research data is unavailable.

### Database and search strategies

We performed a comprehensive search of all the relevant RCTs published by February 2023 in the databases of PubMed, Web of Science, EMBASE, Cochrane CENTRAL, Ovid, China National Knowledge Infrastructure, Chinese BioMedical Literature Database, Wanfang, and VIP Database. Search strategies include the keywords below: “chronic rhinosinusitis”, “endoscopic sinus surgery”, and “enhanced recovery after surgery” (S1 Appendix). We carried out a manual search of the list of references included in this study. In addition, we further searched the Chinese Clinical Trials Registry and ClinicalTrials.gov and considered grey literature to identify relevant eligible literature. We then consulted with information experts in the relevant fields to obtain as much of the potential research as possible and to determine the final search strategy.

### Studies selection

We used EndnoteX9 software to manage the retrieved literature and remove duplicates. Two reviewers (YQW and YQH) independently screened the remaining articles against the eligibility criteria, and then read the selected full text to identify the final included literature. Any differences were resolved in consultation with the third researcher (TMZ).

### Data extraction

Two reviewers (YQW and XRG) performed data extraction based on the pre-designed extraction table. The extracted information included studies ID, disease type, sample size, age in years, course of the disease, ERAS elements, and outcomes. We attempted to contact the authors through email for missing data in the article. In any case of disagreement, we discussed and negotiated with the third reviewer (YJF).

### Assessment of risk of bias

The methodological quality of the selected RCTs was separately assessed by two reviewers (YQW and ZTH) by using the Cochrane risk-of-bias tool [17]. Each project and study was rated as“low risk,” “some concerns” or “high risk”. The quality of the included RCTs was judged based on: randomization process, deviations from intended interventions, missing outcome data, measurement of the outcome, and selection of the reported result. Any disagreement was solved by consulting the third reviewer (HL).

### Meta-analysis

Review Manager version 5.3 software and Stata statistical software Version 12.0 were used for meta-analysis. Dichotomous data were analyzed for outcome by hazard ratio (RR) and 95% confidence intervals (CIs). Mean difference (MD) and 95%CI were calculated for continuous data in the same unit or with the same type of measurement, otherwise, standardized MD (SMD) and 95%CI were applied. If continuous data is presented in median and quartile, we would convert it into mean and standard deviation according to Hozo et al. and Luo et al. [18, 19]. When the heterogeneity was not significant (*I*^2^<50%), we used the fixed-effects model to analyze the effect size; otherwise, the random-effect model was applied. We planned to explore possible factors affecting heterogeneity through subgroup analysis (age [Less than or equal to 45 and greater than 45] and ERAS elements [Less than or equal to 7 and greater than 7]). Potential publication bias was assessed by funnel plots when more than 10 RCTs were included. In addition, Begg’s and Egger’s test were employed as assessment methods for testing. Sensitivity analysis was performed to verify the robustness of the results. Descriptive analysis was adopted when the quantitative analysis is inappropriate (e.g. due to unsynthesizable data).

### Assessment of quality of evidence

The certainty of the evidence was graded by using the Grading of Recommendations Assessment, Development, and Evaluation (GRADE) tool [20]. Evidence outcomes were graded as “high,” “moderate,” “low,” or “very low”, it depends on the evaluation of the risk of bias, inconsistency, indirectness, imprecision, and publication bias. Two reviewers (YQW and HLF) did the evaluation independently and where there was a difference of opinion, attempts were made to discuss or negotiate with a third researcher (HL).

## Results

### Study selection

**Fig 1:**
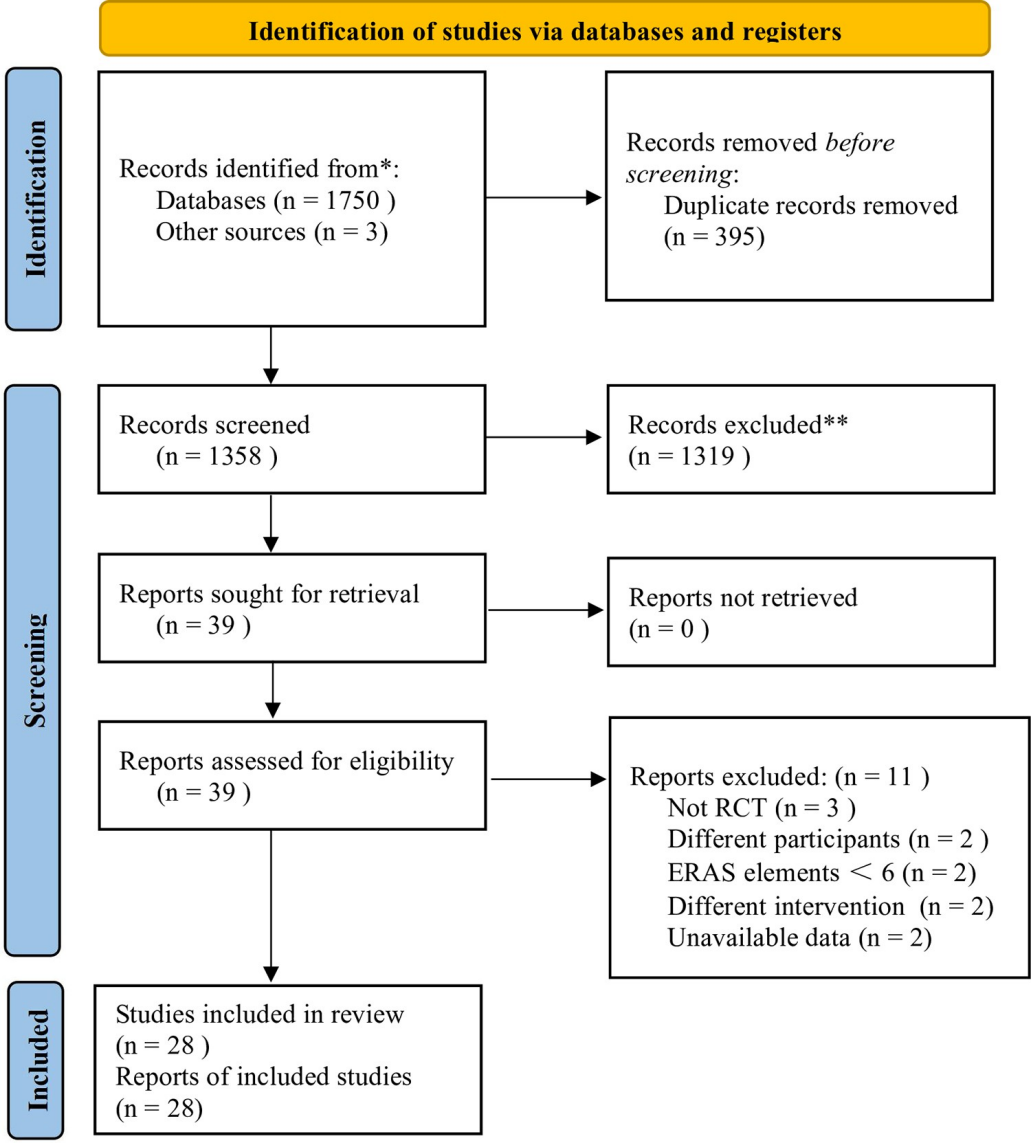
PRISMA flow diagram.

Initially, 1753 potential studies were retrieved, and 395 duplicates were removed. After screening the remaining 1358 articles by title and abstract, 1319 articles were excluded. Then, we read the full text of 39 publications, excluded 11 of them and finally the other 28 studies [5, 16, 21–46] were identified for inclusion in this meta-analysis (Fig 1). The exclusion studies and the relevant reasons are in S2 Appendix.

### Study characteristics

All 28 RCTs enrolled in this meta-analysis involved 2636 CRS patients (1321 in the ERAS group and 1315 in the SC group). 24 studies [21–24, 26–28, 30–46] recruited both CRSwNP and CRSsNP patients, and the other four studies [5, 16, 25, 29] only focused on the patients with CRSwNP. ERAS elements adopted by these studies ranged from 6 to 14. In terms of the research contents, ten studies [16, 21–29] had reported LOS, 13 studies [5, 16, 21, 23, 24, 26, 28–34] had outcomes on the incidence of overall postoperative complication, nine studies [5, 16, 23, 29, 30, 32, 35–37] reported PONV, eight studies [24, 26, 30, 32, 33, 35–37] on facial edema, seven studies [24, 26, 32, 33, 35–37] on low back pain and urinary retention, five studies [21, 23, 29–31] on haemorrhage, 12 studies reported [21, 23, 24, 26, 27, 30–32, 38–41] VAS pain scores, seven studies [30, 31, 34, 42, 43, 45, 46] reported anxiety score, five studies [30, 31, 34, 42, 43] reported depression score, ten studies [5, 28, 31, 33, 37, 39, 41–44] had data on the QOL, and one study [16] reported on hospitalization expenses. The basic characteristics of all the research included were presented in Table 1. The ERAS elements detailed list was shown in Table 2.

**Table 1:** Characteristics of the included studies.

Studies ID	Disease type	Sample Size		Age in years		Course of disease		Intervening measure		Follow-up	ERAS element	Outcomes
		ERAS/female	SC/female	ERAS	SC	ERAS	SC	Study group	control group			
Huang 2020 [21]	CRS	45/19	45/18	35.1±3.7	34.5±3.8	6.2±0.9m	4.2±0.3m	ERAS	SC	-	8	①②③
Liu 2021b [22]	CRS	64/-	64/-	-	-	-	-	ERAS	SC	-	8	①
Shu 2021 [23]	CRS	100/51	100/44	57.1±5.8	56.7±5.1	-	-	ERAS	SC	-	7	①②③
Si 2021 [24]	CRS	60/28	60/29	55.6±5.5	55.6±5.4	2.1±0.3y	2.1±0.3y	ERAS	SC	-	6	①②③
Song 2018 [25]	CRSwNP	70/28	66/30	48.2±11.6	45.1±12.2	-	-	ERAS	SC	-	11	①
Wu 2019b [16]	CRSwNP	52/14	50/11	40.0 ± 10.0	38.8 ± 10.0	-	-	ERAS	SC	-	14	①②⑦
Wu 2021 [26]	CRS	25/10	25/9	55.8±5.5	54.6±5.4	-	-	ERAS	SC	-	6	①②③
Xie 2019 [27]	CRS	32/14	32/12	43.3±5.3	42.5±4.6	5.2±2.5y	4.8±2.2y	ERAS	SC	-	10	③
Yang 2020 [28]	CRS	40/15	40/14	40.5±19.8	41.2±20.3	26.5±15.2m	24.8±16.1m	ERAS	SC	-	11	①②④
Zhan 2021 [29]	CRSwNP	50/22	50/21	40.96±6.24	40.15±6.28	3.34±1.02y	3.34±0.98y	ERAS	SC	3months	6	①②
Fan 2022a [30]	CRS	60/27	60/25	44.23±6.88	43.61±6.27	3.26±0.86y	43.61±6.27y	ERAS	SC	-	10	②③⑤⑥
Fan 2022b [31]	CRS	60/23	60/21	48.64±10.12	49.17±9.51	-	-	ERAS	SC	-	6	②③④⑤⑥
Liu 2022 [32]	CRS	50/22	50/21	55.38±4.82	55.42±4.79	2.63±0.64y	2.61±0.65y	ERAS	SC	-	10	②③
Wu 2019a [5]	CRSwNP	36/-	38/-	-	-	-	-	ERAS	SC	-	12	②④
Yu 2019 [33]	CRS	34/-	34/-	-	-	-	-	ERAS	SC	-	6	②④
Zheng 2020 [34]	CRS	25/9	25/8	41.21±2.21	41.78±2.45	-	-	ERAS	SC	-	7	②⑤⑥
Huang 2019 [35]	CRS	20/11	20/10	44.38±6.04	40.09±5.32	6.5±1.3m	7.5±1.2m	ERAS	SC	-	8	②
Liu 2021a [36]	CRS	35/15	35/17	40.1±23.54	41.2±33.26	-	-	ERAS	SC	-	8	②
Ma 2021 [37]	CRS	60/28	60/24	42.03±5.96	41.36±6.36	-	-	ERAS	SC	-	10	②④
Bai 2021 [38]	CRS	88/42	88/40	37.4±0.84	37.5±0.79	-	-	ERAS	SC	-	7	③
Chen 2022 [39]	CRS	38/16	38/15	43.94±3.26	43.28±3.65	-	-	ERAS	SC	-	6	③④
Han 2022 [40]	CRS	44/13	44/15	48.26±13.07	47.68±13.45	2.93±1.14y	2.70±1.25y	ERAS	SC	-	7	③
Song 2019 [41]	CRS	37/19	37/18	46.33±5.11	46.58±5.05	2.11±0.52y	2.01±0.54y	ERAS	SC	-	8	③④
Guo 2023 [42]	CRS	34/15	34/18	44.12±7.22	46.34±6.32	-	-	ERAS	SC	-	8	④⑤⑥
Hu 2022 [43]	CRS	35/14	35/15	58.91±5.29	59.90±5.24	10.28±2.16y	11.08±2.36y	ERAS	SC	-	7	④⑤⑥
Jin 2020 [44]	CRS	43/24	43/23	46.31±5.39	46.79±5.28	-	-	ERAS	SC	-	6	④
Cao 2021 [45]	CRS	30/12	30/16	37.1±12.3	38.4±8.8	5.2±1.6y	4.9±1.9y	ERAS	SC	7days	8	⑤
Li 2022 [46]	CRS	54/13	52/10	44.73±12.03	44.02±12.48	-	-	ERAS	SC	1week	8	⑤

Note:①length of stay ②complications ③VAS score ④quality of life ⑤anxiety symptoms ⑥depressive symptoms ⑦hospitalization expenses

CRS, chronic rhinosinusitis; CRSwNP, chronic rhinosinusitis with nasal polyps; ERAS, enhanced recovery after surgery; SC, standard care; m, months; y, years.

**Table 2:** Summary of ERAS elements.

Period	ERAS items	Huang 2020 [21]	Liu 2021b [22]	Shu 2021 [23]	Si 2021 [24]	Song 2018 [25]	Wu 2019b [16]	Wu 2021 [26]	Xie 2019 [27]	Yang 2020 [28]	Zhan 2021 [29]	Yu 2019 [33]	Chen 2022 [39]	Fan 2022a [30]	Zheng 2020 [34]
Pre-op	Preoperative education and counseling		√	√	√	√	√	√	√	√	√	√	√	√	√
	Preoperative airway management					√				√					
	Preoperative standardized medication					√	√								
	Antimicrobial prophylaxis		√			√	√	√							
	Preoperative analgesia					√	√		√						
	Preoperative fluid management	√					√		√	√				√	√
	Preoperative training	√	√	√	√			√		√		√	√	√	
	Pre-operative fasting (oral)		√	√	√		√		√	√	√	√	√	√	√
	Optimal anesthesia scheme					√	√		√						
Intra-op	Intraoperative analgesia						√								
	Intraoperative fluid management					√	√		√					√	
	Appropriate surgical procedure (nasal tamponade)					√	√		√	√					
	Maintenance of normothermia	√				√	√			√				√	
Post-op	Postural care	√	√	√	√			√		√	√	√	√	√	√
	Postoperative fluid management	√	√									√		√	
	Early feeding	√	√	√	√	√	√	√	√	√	√	√	√	√	√
	Early Mobilization	√		√			√		√	√	√				√
	postoperative analgesia	√	√	√	√	√	√	√	√	√	√		√	√	√
Period	ERAS items	Fan 2022b [31]	Liu 2022 [32]	Wu 2019 a [5]	Huang 2019 [35]	Liu 2021a [36]	Ma 2021 [37]	Cao 2021 [45]	Guo 2023 [42]	Hu 2022 [43]	Li 2022 [46]	Jin 2020 [44]	Song 2019 [41]	Han 2022 [40]	Bai 2021 [38]
Pre-op	Preoperative education and counseling	√	√	√		√		√	√	√	√	√	√	√	√
	Preoperative airway management								√						
	Preoperative standardized medication						√								
	Antimicrobial prophylaxis						√							√	
	Preoperative analgesia			√											
	Preoperative fluid management		√	√	√	√	√	√		√	√	√	√		
	Preoperative training	√	√		√	√	√	√			√		√	√	√
	Pre-operative fasting (oral)	√	√	√	√		√	√	√	√	√	√	√		√
	Optimal anesthesia scheme			√					√						
Intra-op	Intraoperative analgesia			√											
	Intraoperative fluid management		√	√				√	√						
	Appropriate surgical procedure (nasal tamponade)			√											
	Maintenance of normothermia		√	√	√	√	√	√				√		√	√
Post-op	Postural care	√	√		√	√	√			√	√		√	√	√
	Postoperative fluid management		√		√	√	√						√		
	Early feeding	√	√	√	√	√	√	√	√	√	√		√	√	√
	Early Mobilization			√				√	√	√	√	√			
	postoperative analgesia	√	√	√	√	√	√		√	√	√	√	√	√	√

### Assessment of risk of bias

**Fig 2:**
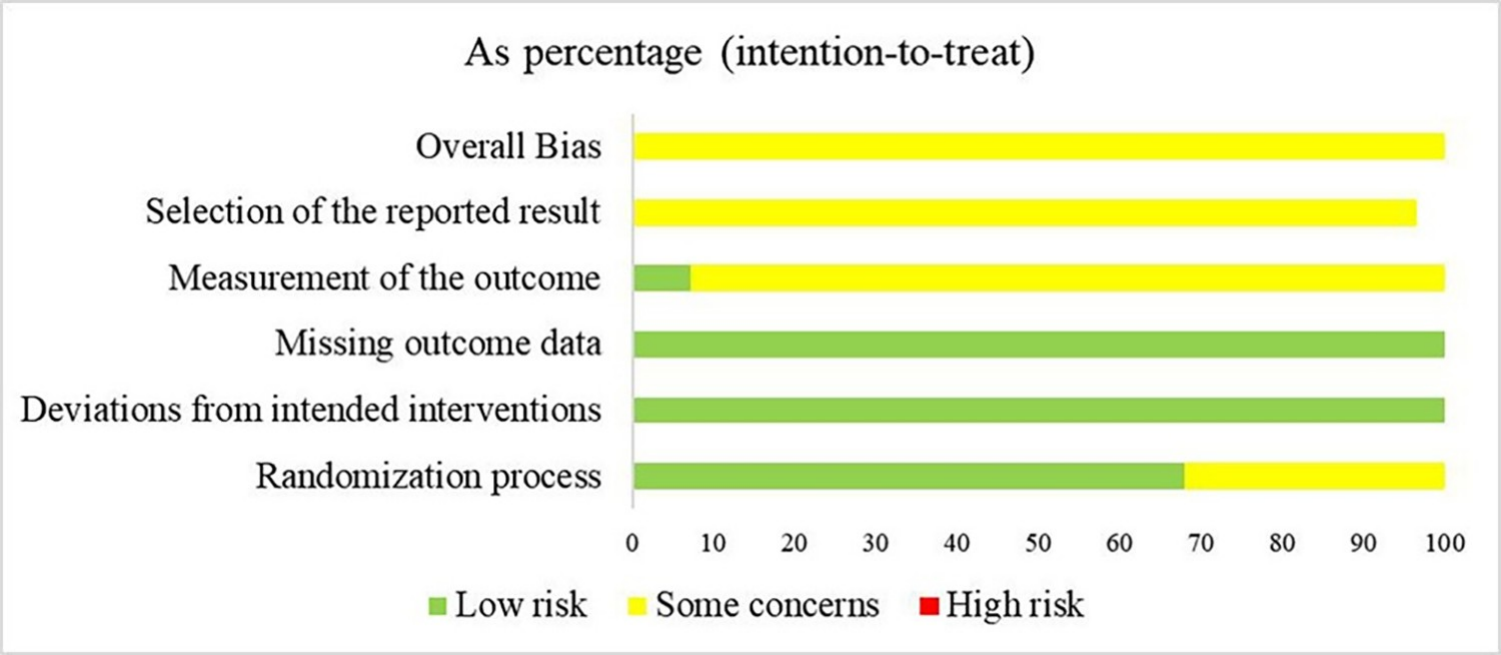
Risk of bias graph.

**Fig 3:**
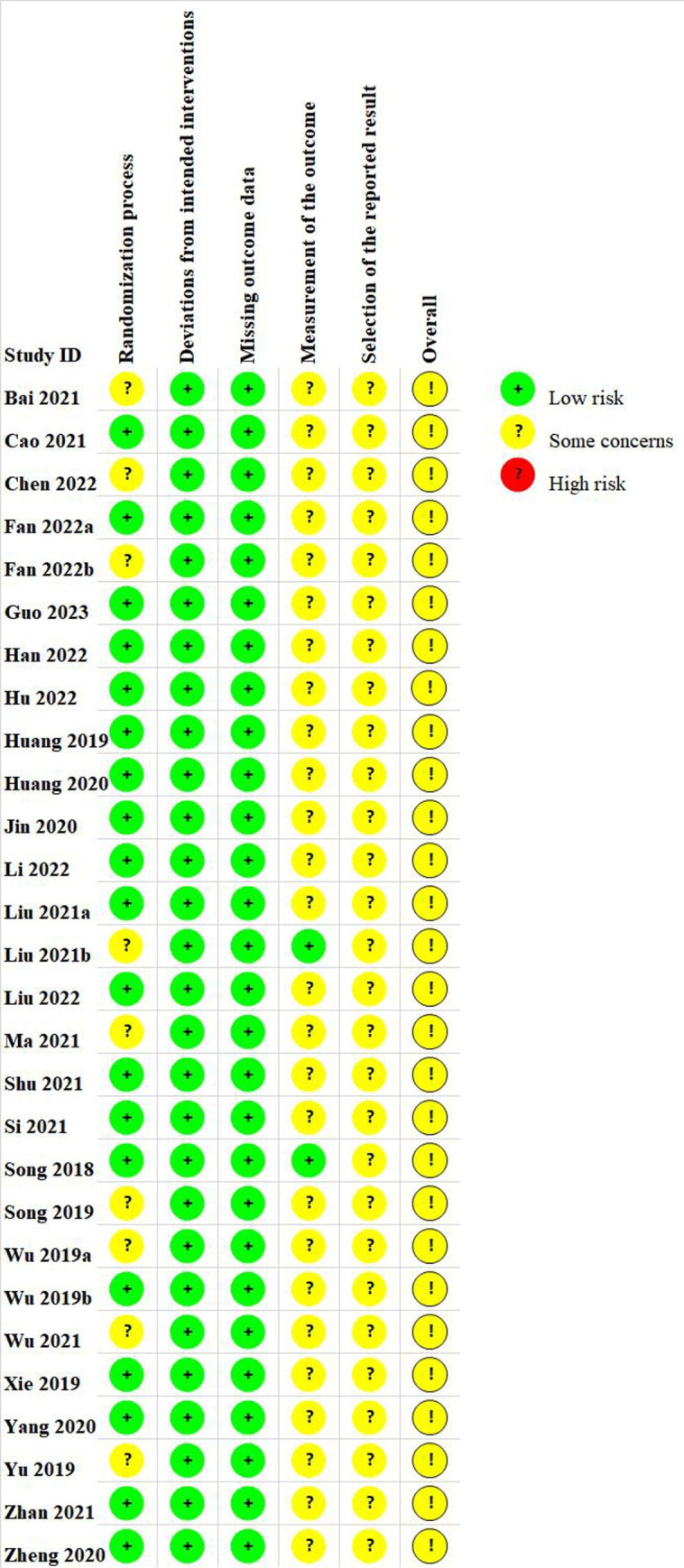
Risk of bias summary.

Among the 28 RCTs, 19 studies [16, 21, 23–25, 27–30, 32, 34–36, 40, 42–46] reporting appropriate randomization methods such as random number tables, random lottery, and so on, were rated low risk. The other nine studies [5, 22, 26, 31–33, 37–39, 41] only indicated the use of randomization and did not describe specific assigned hidden information, so the associated risk of bias was listed as some concerns. The baseline data of all the studies were comparable. Due to the experimental design, it may have been difficult to blind participants and personnel in the studies. All 28 studies had no missing or biased data so they were rated as low risk of deviations from the intended intervention and of missing outcome data. Additionally, no study mentioned the blind method of outcome evaluators. Among them, 26 studies [5, 16, 21, 23, 24, 26–46] scoring the measurement of outcome on a subjective scale were rated as the risk of some concerns, whereas the other two studies [22, 25] with an objective presentation of outcomes were scored as low risk. All 28 studies were evaluated as some concerns due to selective reporting of results (with no protocol or partial protocol). (Figs 2 and 3)

## Primary outcome

### LOS

**Fig 4:**
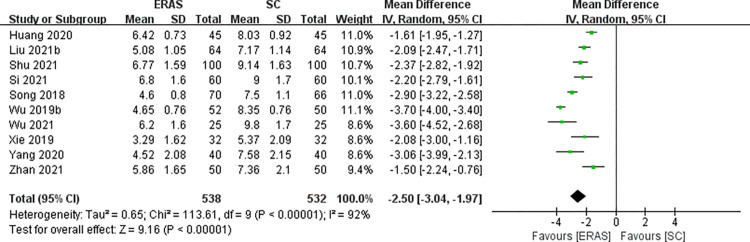
The funnel plot of LOS.

Ten studies (totaling 1070 CRS patients) [16, 21–29] reported LOS. Heterogeneity among studies was high (*I*^2^ = 92%), and a random effects model was adopted. The results displayed that LOS was considerably shorter in the ERAS versus SC group (MD = -2.50, 95%CI: -3.04 to -1.97) (Fig 4). To investigate potential factors that may influence heterogeneity, we performed subgroup analysis by age and ERAS elements. Despite that, the results of the subgroup analysis still had great heterogeneity, indicating that heterogeneity may be derived from other sources (S3 Appendix).

## Secondary outcomes

### Complications

**Fig 5:**
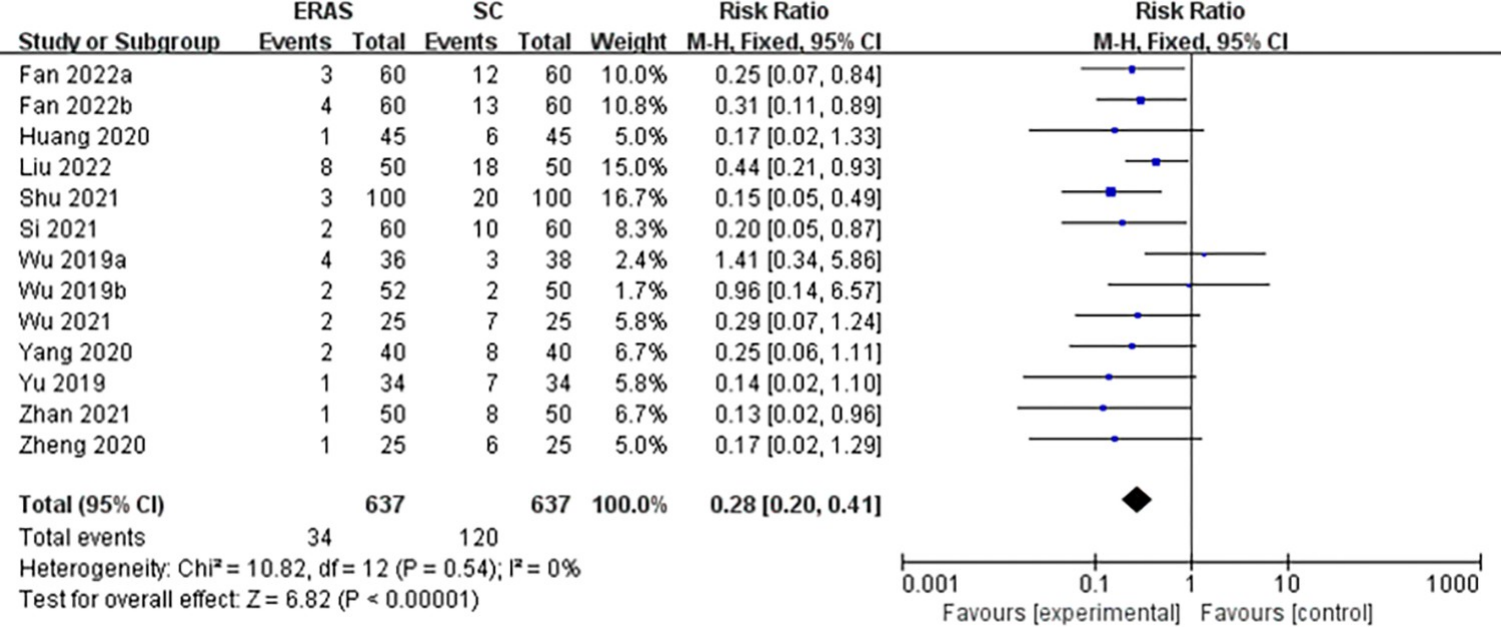
The funnel plot of overall complications.

**Fig 6:**
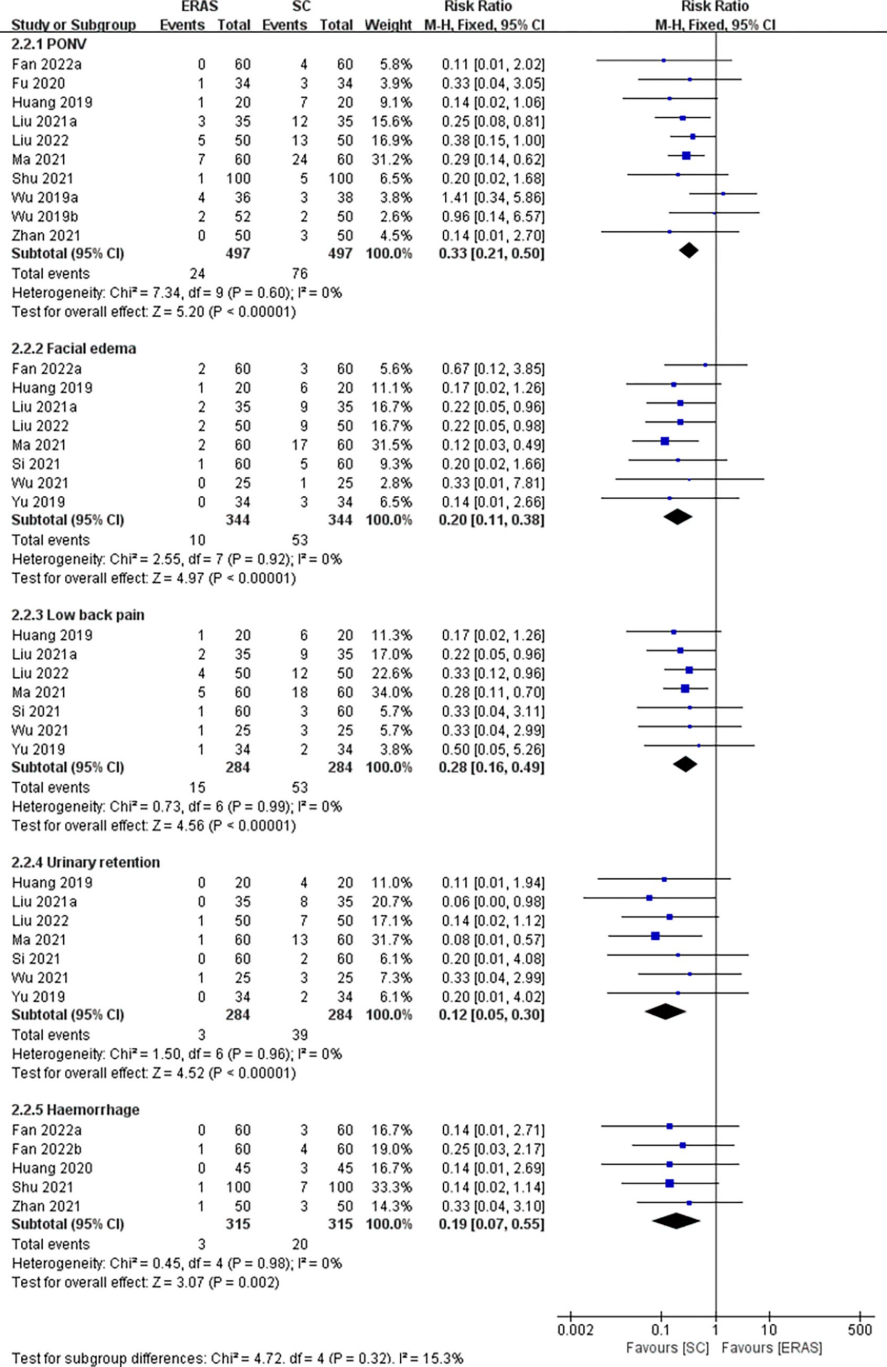
The funnel plot of complications.

Thirteen studies (a total of 1274 patients) [5, 16, 21, 23, 24, 26, 28–34] reported overall postoperative complications. Specifically, 9 studies [5, 16, 23, 29, 30, 32, 35–37] reported PONV (involving 926 CRS patients), 8 studies [24, 26, 30, 32, 33, 35–37] for facial edema (involving 688 CRS patients), 7 studies [24, 26, 32, 33, 35–37] for low back pain and urinary retention (involving 568 CRS patients), and 5 studies [21, 23, 29–31] for haemorrhage (involving 630 CRS patients). These studies had no significant heterogeneity (*I*^2^ = 0%), so meta-analysis was conducted with the fixed-effects model. Our findings indicated that in comparison with the SC group, CRS patients in the ERAS group had a relatively lower complication rate, manifesting as overall complications (RR = 0.28, 95%CI:0.20 to 0.41), PONV (RR = 0.33, 95%CI:0.21 to 0.50), facial edema (RR = 0.20, 95%CI:0.11 to 0.38), low back pain (RR = 0.28, 95%CI:0.16 to 0.49), urinary retention (RR = 0.12, 95%CI:0.05 to 0.30), and haemorrhage (RR = 0.19, 95%CI:0.07 to 0.55) (Figs 5 and 6).

### VAS pain score

**Fig 7:**
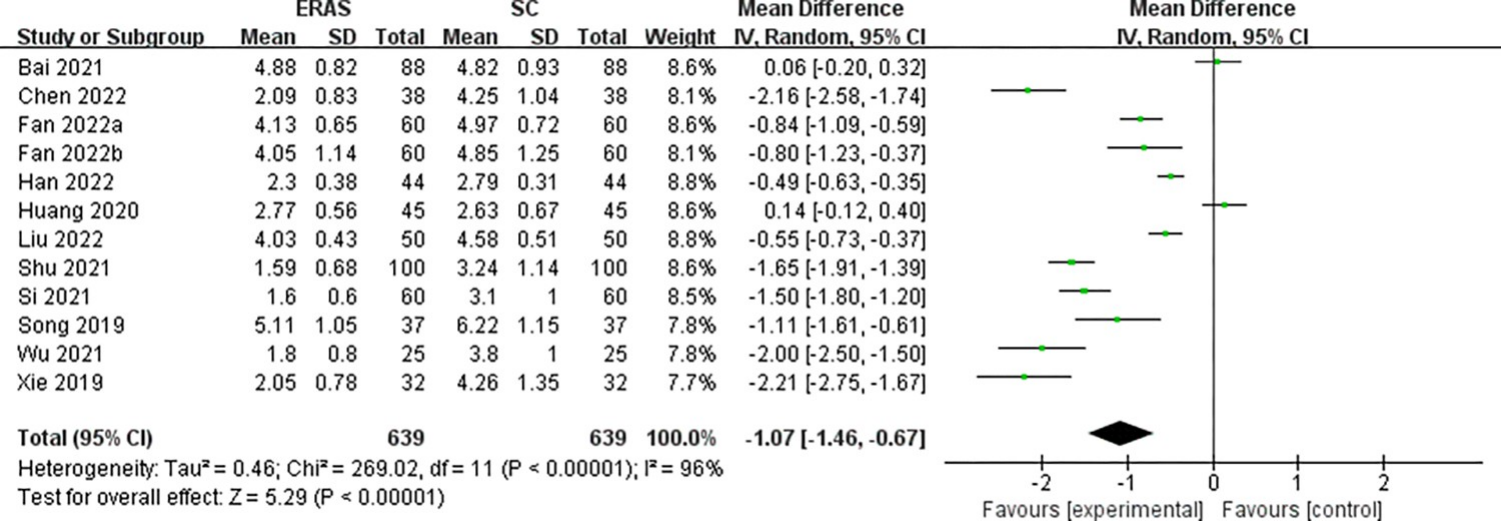
The funnel plot of VAS pain score.

Twelve studies (a total of 1278 patients) [21, 23, 24, 26, 27, 30–32, 38–41] reported VAS pain scores. There was remarkable heterogeneity across studies (*I*^2^ = 96%), so we performed analysis with a random-effects model. Meta-analysis demonstrate that ERAS can effectively relieve pain compared to the SC group (MD = -1.07, 95%CI: -1.46 to -0.67) (Fig 7). Furthermore, no potential sources of heterogeneity were identified in the subgroup analysis based on age and elements (S4 Appendix).

### Anxiety and depression score

**Fig 8:**
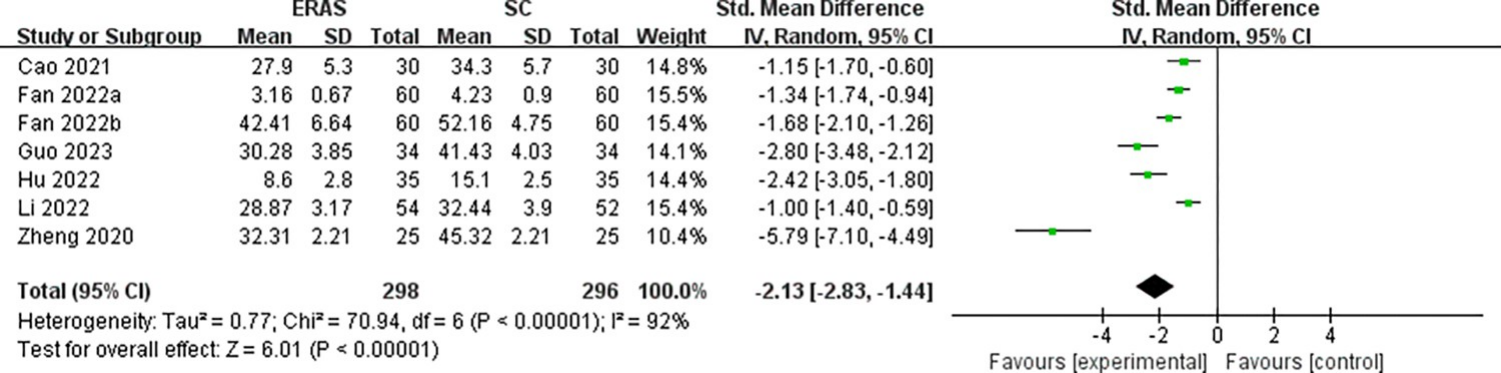
The funnel plot of anxiety score.

**Fig 9:**
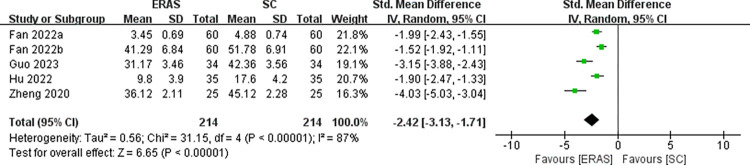
The funnel plot of depression score.

Seven RCTs [30, 31, 34, 42, 43, 45, 46] and five RCTs [30, 31, 34, 42, 43], involving 594 and 428 patients, reported anxiety score and depression score, respectively. Among the anxiety results, 5 studies [31, 34, 42, 45, 46], 1 study [43] and 1 study [30] reported using SAS scale, HAMA scale and GAD-7 scale to evaluate the anxiety score respectively. Among depression outcomes, 3 studies [31, 34, 42], 1 study [43], and 1 study [30] reported using the SDS scale, HAMD scale, and PHQ-9 scale to assess depression scores, respectively. Due to the large heterogeneity (*I*^2^ = 92% and *I*^2^ = 87%), a random effects model was appropriate. Meta-analysis indicated that ERAS group had much lower anxiety score (SMD = -2.13, 95%CI: -2.83 to -1.44) and depression score (SMD = -2.42, 95%CI: -3.13 to -1.71) than the SC group, respectively (Figs 8 and 9). Heterogeneity was not reduced by age and ERAS elements subgroup analysis, suggesting that neither aspect was a potential source of heterogeneity (S5 and S6 Appendices).

## Publication bias

We conducted Begg’s test, Egger’s test, and funnel plot test on the results of ten or more studies. No evident publication bias regarding LOS, overall complications, and VAS pain score was discovered (S7 Appendix).

### Sensitivity analysis

The robustness of our findings was verified by a sensitivity analysis conducted by removing studies one by one (S8 Appendix).

## Assessment of evidence quality

We further assessed the meta-results of GRADE quality evidence. The quality of evidence of overall postoperative complications, PONV, facial edema, low back pain, urinary retention, and haemorrhage was considered “moderate”, while the remaining outcomes were rated as “very low”. Detailed results can be found in S9 Appendix.

### Descriptive analysis

Descriptive analyses were also performed in this study to supplement the outcomes for which quantitative meta-analysis was not successful. Among the included studies, ten studies [5, 28, 31, 33, 37, 39, 41–44] reported patients’ QOL from different evaluation criteria. We were unable to combine the data due to opposite evaluation criteria, so descriptive analysis was used. Eight studies [31, 33, 37, 39, 41–44] used a QOL scale that was negatively correlated with disease severity, with higher scores being accompanied by better QOL and less severe disease severity. Three studies [37, 41, 44] used the GQOL-74 scale, four studies [31, 33, 39, 42] used a homemade life scale, and one study [43] used the EORTC core quality of life questionnaire (QLQ-C30) scale, and all eight studies yielded significantly higher QOL scores in the postoperative ERAS versus SC group (*P* < 0.05), reflecting the improved QOL of postoperative patients in the ERAS group and the effectiveness of the comprehensive nursing measures for rehabilitation. In addition to this, two other studies [5, 28] used a QOL scale that was positively correlated with disease severity, with higher scores being associated with poorer quality of life and more severe disease. In one study [28], patients’ QOL was evaluated using the SNOT-20 scale, and it was observed that the QOL scores of CRS patients of the ERAS group were markedly reduced compared to the SC group after surgery (*P* < 0.05), which reflected a meaningful enhancement in the QOL of the patients. Wu et al. [5] evaluated the QOL scores of CRS patients preoperatively, and postoperatively on the first, third, and sixth days by using the SNOT-22 scale, which showed that the QOL scores of CRS patients in the ERAS group were significantly lower compared to those of the SC group on the first and third postoperative days (*P* < 0.05). However, the QOL scores were on the high side in both groups compared to baseline. This reflects that the degree of recovery in the ERAS group was better when compared with the SC group, but the patients’ QOL had not yet returned to the preoperative level in the postoperative period of 3 days, implying that the patients had not yet received complete and effective recovery and relief; It is noticeable that the QOL scores returned to levels below baseline on the sixth postoperative day in both groups, but did not reflect a difference between the two groups. This suggests that the recovery of overall symptoms and psychological conditions of the patients was more significant and productive on the sixth postoperative day, but the long-term impact on QOL in both groups needs to be observed with more follow-up; Wu et al. [5] also observed that CRS patients in the ERAS group recovered better than the SC group in terms of rhinology and sleep function, suggesting that rhinology and sleep function appear to be the main contributors to quality of life.

It is well known that quality of life, as a dynamic evaluation criterion, is important to understand the degree of relief of patients’ symptoms, the effectiveness of care and the degree of recovery from rehabilitation [47]. Quality of life evaluation can reflect the degree of symptomatic improvement and recovery of CRS patients in the perioperative period, which is an important evaluation standard of the comprehensive severity of the patient’s physical or psychological. Therefore, more studies may be needed to evaluate the QOL in CRS patients after perioperative implementation of ERAS protocols.

Only one study [16] reported hospitalization expenses, and we used descriptive analyses because the criteria that allowed for quantitative synthetic analyses of the data were not met. Wu et al. [16] reported that the hospitalization expenses of CRS patients in the ERAS group were notably less than the SC group(*P*<0.001), and the study also found that there were shorter LOS in the ERAS group. In summary, the limited evidence suggests that hospitalization expenses are an endpoint of LOS and that they are positively correlated. In conclusion, the integrated care model in the ERAS protocol facilitated the great reduction of LOS for CRS patients, which contributed to a reduction in hospitalization expenses. However, more study evidence is in demand to prove this in the future.

As for other outcomes not included in this study, seven studies [27, 29, 31, 34, 42, 43, 46] reported that the implementation of an ERAS protocol increased overall perioperative satisfaction in CRS patients; four studies [28, 32, 41, 44] reported significant improvements in olfactory function and nasal resistance in CRS patients in the ERAS group; two studies [16, 46] reported that an ERAS protocol could significantly increase perioperative comfort in CRS patients. These results demonstrate the effectiveness of the ERAS protocol for perioperative recovery in CRS patients from other perspectives.

Obviously, from both a subjective and objective point of view, the adoption of the ERAS protocol throughout the entire ESS period in CRS patients has been shown to be favorable for the earliest and quickest possible recovery of the patients when compared with the SC group, which highlights the necessity of the clinical applicability of the ERAS protocol in the ESS perioperative period of CRS patients.

## Discussion

ERAS is an innovative perioperative care mode, which breaks through the traditional surgical nursing mode [48, 49]. This emerging recovery management mode covers preoperative education and counseling, fluid management, multimodal analgesia, early feeding and mobilization, etc. Until now, there is a limited comprehensive understanding of the efficacy of ERAS protocols in perioperative CRS patients. In this review, we compared the impacts of ERAS with SC protocols on CRS patients undergoing ESS by a comprehensive analysis of relevant RCTs. Our results show that the ERAS group is associated with shorter total LOS, lower complication rates, reduced postoperative pain, reduced anxiety and depression, reduced hospitalization expenses, and improved patient QOL versus the SC group. On the whole, in terms of the efficacy and safety of care strategies, ERAS has a distinct advantage over conventional standard care. These included researches drew a consistent conclusion that ERAS could contribute to physical and psychological recovery in CRS patients after ESS. Sensitivity analysis confirmed the robustness of our findings. However, there existed inevitably some heterogeneity in the evaluation outcomes of the included study. To detect factors influencing heterogeneity, we performed subgroup analysis for highly heterogeneous outcomes, whereas subgroup typing by age and ERAS elements did not eliminate the heterogeneity of the above-mentioned evaluation outcomes. We think such heterogeneity is probably associated with the difference of ERAS elements implementation or evaluation approaches in the included studies.

LOS is a crucial indicator for measuring the effectiveness of integrated care ERAS protocols. In this study, either pooled or subgroup analyses strongly suggested that ERAS management after ESS could reduce the LOS of CRS patients effectively, which is congruous with the reported findings from other meta-analysis research on ERAS protocols including bariatric surgery by Zhou et al [15], cesarean section by Meng et al [11], renal tumor resection by Wu et al [50], and pancreaticoduodenectomy by Cao et al [51]. The above-mentioned research demonstrates that patients receiving ERAS mode could often be discharged from the hospital earlier than that receiving conventional care. In the ERAS program, preoperative counseling, preoperative training, effective postoperative analgesia, early feeding, and early mobilization have a positive action on shortening the duration of hospitalization. It is well-known that CRS not only damages the patient’s health but also brings severe financial burdens on their families [6]. There is no doubt that shortened LOS is beneficial to reducing hospitalization expenses. Our findings also provided evidence that ERAS management could cut down on hospitalization costs for CRS patients undergoing ESS. Notably, there is just one report in our study involving hospitalization expenses. As a consequence, such a result should be interpreted with caution, and the impacts of ERAS management on CRS patients’ hospitalization costs need to be further illustrated with more large-sample studies.

The occurrence of postoperative complications is an extremely powerful marker for visualizing the safety of ERAS. Complications tend to prolong patients’ hospital stays and increase costs, which have become a major problem in postoperative care. In some non-sinus operations, ERAS protocols have displayed superiority in improving postoperative complications [10, 11]. Our findings similarly indicated that ERAS management significantly declined the rate of postoperative complications in CRS patients. Preoperative education and counseling, fluid management, optimization of anesthetic modalities, appropriate nasal tamponade, minimally invasive surgical manipulation by the surgeon, and reduced opioid usage in the postoperative period in ERAS were possibly associated with reduced nausea and vomiting, facial edema, haemorrhage, and urinary retention; in addition, postural care and early mobilization may be associated with reduced low back pain. The implementation of ERAS enables the alleviation of the surgery-induced stress response, effectively minimizes the occurrence of complications, and facilitates the early rehabilitation of patients, thus further reducing the LOS and hospitalization costs and improving the QOL of patients.

Pain has an important influence on patients’ postoperative recovery. For perioperative patients, part of the vital care target is to relieve pain. Pain management is a key component of ERAS protocols. Other meta-analyses suggested that ERAS protocols could provide effective pain management for patients [11, 52, 53]. There are 4 studies for preoperative analgesia management, 2 studies for intraoperative pain management, and 26 studies for postoperative pain management included in our research. We found that CRS patients from the ERAS group scored distinctly lower pain than patients from the SC group. Besides that, Gao et al.’s [54] prospective cohort study surveyed pain in CRS patients undergoing ESS and found a dramatic improvement in pain after 6 hours postoperatively in patients of ERAS group. Multimodal analgesia in ERAS strategies provides optimal pain relief for CRS patients, reducing the use of opioids and their harm to body organs, allowing early-stage feeding and early mobilization for patients after surgery as well as enabling a higher QOL [16, 55]. It is seen clearly that ERAS strategies can provide effective and safe pain management for CRS patients, and further promote rapid function restoration in postoperative CRS patients.

All patients’ discomforts tend to be caused by both physical and psychological factors. The previous study indicated clearly that the severity of CRS could be related to the psychological disorders of patients [55]. Psychological disorders such as anxiety and depression might amplify CRS patients’ perception of pain to some extent, affecting the prognosis as well as QOL [3]. To avoid these mentioned problems, it is imperative to focus on the mental health of CRS patients in the perioperative period. The cohort study by Gao et al [54] suggested that ERAS program could improve the emotions of CRS patients who receive ESS. In this meta-analysis, anxiety and depression scores as well as pain scores were markedly lower in ERAS versus SC group. It is seen that ERAS strategies could facilitate in eliminating negative emotions of perioperative patients, and further alleviate the pain.

Regarding the impact of ERAS on patient QOL, nine of the included studies demonstrate a beneficial effect of ERAS on improving QOL in CRS patients undergoing ESS. However, the study by Wu et al [5] presented that QOL was not appreciably dissimilar between the ERAS and SC groups at postoperative day 6. Given the absence of follow-up data on patient QOL in the included studies, caution needs to be made when interpreting QOL results. Also, the effect of ERAS on patient QOL needs to be validated by a large follow-up trial.

ERAS is considered an evidence-based perioperative care mode. There have been many explanations for the effects of the items in ERAS protocols on the prognosis of the patients. Education, counseling and training before the operation can diminish anxiety and other negative feelings, build patients’ confidence in recovery, and increase treatment compliance of patients [55, 56]. Shortening the time of pre-surgical fasting and water deprivation helps to accelerate the rehabilitation rate of patients. Preoperative carbohydrate loading has been reported to attenuate operation stress, protein loss as well as insulin resistance and is beneficial in maintaining body weight and muscle strength [57, 58]. Preoperative airway management can effectively prevent postoperative trauma response and lung injury [59]. Maintaining perioperative fluid balance can reduce intravascular fluid transfer and optimize cardiac output [57]. Keeping body temperature can reduce not only oxygen consumption but also the risk of complications [59, 60]. Prophylactic use of antimicrobial agents could remarkably decrease the incidence of postoperative infection [59]. Optimized programs of anesthesia and perioperative analgesia could ease metabolic stress responses, promote rapid awakening and efficaciously relieve pain [55]. Early-stage feeding and mobilization after surgery can also help to counteract brake-induced insulin resistance and improve muscle strength [59, 61]. Posture nursing could alleviate low back pain caused by staying in one position for long periods [41]. Our study demonstrated the effectiveness and safeness of ERAS programs for perioperative CRS patients undergoing ESS while providing first-hand evidence for early design of the specialized ERAS guidelines for ESS. Additionally, the formulation of ERAS protocols should be tailored to the actual situation of patients. Only a suitable combination of the ERAS items can greatly reduce operation stress response and recovery time.

ERAS in a wide range of diseases has gained approval and support from many international guidelines, and it has been widely applied with encouraging treatment results [62–64]. However, the ERAS protocol is a newly developed and emerging model of care in the field of CRS, for which there is no international guideline guidance to date in this area, and for which the development of ERAS is currently at an incomplete stage of maturity in CRS. Therefore, it is unfortunate that the ERAS concept has not yet been fully popularized in the mind of every ENT surgeon, which may lead to the fact that most surgeons in the clinic tend to focus only on surgical techniques when performing surgery on CRS patients, neglecting the overall nursing management of the patient’s psychological and physiological aspects during the whole perioperative period, which may affect the physical and mental recovery of the patient’s rehabilitation. In future medical work, efforts should be made to popularize ERAS protocol in the field of otolaryngology, so that medical personnel in all departments of otolaryngology are fully aware of the possible clinical benefits of ERAS, which will lead to further implementation and development of ERAS within the perioperative period of CRS patients. It is worth noting that the implementation of a single ERAS element often does not result in a significant clinical benefit, but with the active efforts of multiple teams and multiple elements combined, we’ll reap much more benefits from the synergies [65]. This implies the importance of teamwork and the need to gain the patient’s understanding. Only in this way can we break free from traditional nursing concepts and realize great perioperative clinical benefits, thus improving patient recovery rates.

Another key element to consider is the importance of the clear development of relevant guidelines, which will give a well-defined standardized model for administering ERAS protocols in the ESS perioperative period of CRS, thus facilitating the further development of ERAS protocols in the field of CRS. In the development of future ERAS clinical guidelines on the perioperative period for CRS patients, the focus should be on elements specific to CRS perioperative care that should be attended to, particularly nasal care, which may be more favorable for surgical recovery in CRS. This review contributed positive and beneficial evidence support for the adoption of ERAS protocols in the field of CRS, but the included studies were limited and suffered from unavoidable study quality deficiencies. Therefore, more high-quality and rigorous RCTs in the future have to be conducted to validate the effectiveness and safeness of applying ERAS protocols in the ESS perioperative period in CRS patients.

In order to ensure the integrity and reliability of this study, our group conducted a comprehensive search of the RCTs in previously mentioned databases. However, there are some inevitable limitations to this review. Firstly, the overall risk of bias for the inclusion studies in this review was some concerns, which may have biased the results. Secondly, ERAS protocols adopted by the included studies had some differences and there was high heterogeneity in the study results, which may have led to biased results. Thirdly, the included studies lacked follow-up data and did not reflect the long-term efficacy of ERAS. Fourthly, only studies published in English and Chinese were included in this study, which might introduce some language bias. Finally, due to the different disease categories of CRS patients enrolled in the study, there may be some differences in disease severity, which may contribute to some bias in our results.

## Conclusion

This research indicated that, for CRS patients undergoing ESS, ERAS protocols could contribute to reduction of LOS and hospitalization expenses, relief of pain, elimination of negative feelings such as anxiety and depression, prevention of the complications and improvement of QOL. The current research results initially demonstrated the effectiveness and safeness of ERAS protocols for perioperative CRS patients, in favor of the further popularization of such rehabilitation protocols in clinical practice. However, we might just see the tip of the iceberg in this field. To address the problems of heterogeneity and risk of bias, more large-sized and high-quality studies should be conducted in the future.

## Supporting information

S1 Appendix Database and search strategies. (DOC)

S2 Appendix Excluded literature and reasons. (DOC)

S3 Appendix Subgroup analysis of LOS. (DOC)

S4 Appendix Subgroup analysis of VAS pain score. (DOC)

S5 Appendix Subgroup analysis of anxiety score. (DOC)

S6 Appendix Subgroup analysis of depression score. (DOC)

S7 Appendix Publication bias. (DOC)

S8 Appendix Sensitivity analysis. (DOC)

S9 Appendix Assessment of evidence quality. (DOC)

S10 Appendix PRISMA 2020 checklist. (DOCX)
