# Supplementary material for: The application of enhanced recovery after surgery (ERAS) in chronic rhinosinusitis patients undergoing endoscopic sinus surgery: A systematic review and meta-analysis
Source: PLoS One. 2023 Sep 21;18(9):e0291835. doi: 10.1371/journal.pone.0291835 (PMC10513253; doi:10.1371/journal.pone.0291835)
Supplement: S1 Appendix — (DOC) [file pone.0291835.s001.doc]

**S1 Appendix. Database and Search Strategies**

**PubMed**

|  | Searches | Results |
| --- | --- | --- |
| #12 | ((("Sinusitis"[Mesh]) OR (((((((((sinusitis [Title/Abstract]) OR (rhinosinusitis [Title/Abstract])) OR (chronic sinusitis [Title/Abstract])) OR (chronic rhinosinusitis[Title/Abstract])) OR (nasosinusitis [Title/Abstract])) OR (paranasal sinus disease[Title/Abstract])) OR (nasal polyp[Title/Abstract])) OR (sinus*[Title/Abstract])) OR (CRS[Title/Abstract]))) AND ((("Nasal Surgical Procedures"[Mesh]) OR ("Otorhinolaryngologic Surgical Procedures"[Mesh])) OR (((((endoscopic sinus surgery [Title/Abstract]) OR (polypectomy [Title/Abstract])) OR (sinus surgery [Title/Abstract])) OR (FESS[Title/Abstract])) OR (ESS[Title/Abstract])))) AND ((("Perioperative Care"[Mesh]) OR ("Enhanced Recovery After Surgery"[Mesh])) OR (((((((Perioperative Care[Title/Abstract]) OR (enhanced recovery after surgey[Title/Abstract])) OR (enhanced recovery pathway[Title/Abstract])) OR (fast track surgery[Title/Abstract])) OR (fast track recovery[Title/Abstract])) OR (ERAS[Title/Abstract])) OR (FTS[Title/Abstract]))) | 489 |
| #11 | (("Perioperative Care"[Mesh]) OR ("Enhanced Recovery After Surgery"[Mesh])) OR (((((((Perioperative Care[Title/Abstract]) OR (enhanced recovery after surgey[Title/Abstract])) OR (enhanced recovery pathway[Title/Abstract])) OR (fast track surgery[Title/Abstract])) OR (fast track recovery[Title/Abstract])) OR (ERAS[Title/Abstract])) OR (FTS[Title/Abstract])) | 167933 |
| #10 | (("Nasal Surgical Procedures"[Mesh]) OR ("Otorhinolaryngologic Surgical Procedures"[Mesh])) OR (((((endoscopic sinus surgery [Title/Abstract]) OR (polypectomy [Title/Abstract])) OR (sinus surgery [Title/Abstract])) OR (FESS[Title/Abstract])) OR (ESS[Title/Abstract])) | 120550 |
| #9 | ("Sinusitis"[Mesh]) OR (((((((((sinusitis [Title/Abstract]) OR (rhinosinusitis [Title/Abstract])) OR (chronic sinusitis [Title/Abstract])) OR (chronic rhinosinusitis[Title/Abstract])) OR (nasosinusitis [Title/Abstract])) OR (paranasal sinus disease[Title/Abstract])) OR (nasal polyp[Title/Abstract])) OR (sinus*[Title/Abstract])) OR (CRS[Title/Abstract])) | 203798 |
| #8 | ((((((Perioperative Care[Title/Abstract]) OR (enhanced recovery after surgey[Title/Abstract])) OR (enhanced recovery pathway[Title/Abstract])) OR (fast track surgery[Title/Abstract])) OR (fast track recovery[Title/Abstract])) OR (ERAS[Title/Abstract])) OR (FTS[Title/Abstract]) | 13525 |
| #7 | ((((endoscopic sinus surgery [Title/Abstract]) OR (polypectomy [Title/Abstract])) OR (sinus surgery [Title/Abstract])) OR (FESS[Title/Abstract])) OR (ESS[Title/Abstract]) | 20983 |
| #6 | ((((((((sinusitis [Title/Abstract]) OR (rhinosinusitis [Title/Abstract])) OR (chronic sinusitis [Title/Abstract])) OR (chronic rhinosinusitis[Title/Abstract])) OR (nasosinusitis [Title/Abstract])) OR (paranasal sinus disease[Title/Abstract])) OR (nasal polyp[Title/Abstract])) OR (sinus*[Title/Abstract])) OR (CRS[Title/Abstract]) | 200538 |
| #5 | "Enhanced Recovery After Surgery"[Mesh] | 1322 |
| #4 | "Perioperative Care"[Mesh] | 158194 |
| #3 | "Otorhinolaryngologic Surgical Procedures"[Mesh] | 100867 |
| #2 | "Nasal Surgical Procedures"[Mesh] | 12214 |
| #1 | "Sinusitis"[Mesh] | 22919 |

**Web of Science**

|  | Searches | Results |
| --- | --- | --- |
| #4 | #1 AND #2 AND #3 | 10 |
| #3 | TS = ("Perioperative Care" OR "Enhanced Recovery After Surgery" OR "enhanced recovery pathway" OR "fast track surgery" OR "fast track recovery" OR "ERAS" OR "FTS") | 15144 |
| #2 | TS = ("Otorhinolaryngologic Surgical Procedures" OR "nasal surgical procedures" OR "endoscopic sinus surgery" OR "polypectomy" OR "sinus surgery" OR "FESS" OR "ESS") | 17687 |
| #1 | TS = ("sinusitis" OR "rhinosinusitis" OR "chronic rhinosinusitis" OR "chronic sinusitis" OR "nasosinusitis" OR "paranasal sinus disease" OR "nasal polyp" OR "sinus*" OR "CRS") | 102344 |

**Embase**

|  | Searches | Results |
| --- | --- | --- |
| #17 | #14 AND #15 AND #16 | 846 |
| #16 | #8 OR #9 OR #10 OR #13 | 182360 |
| #15 | #5 OR #6 OR #7 OR #12 | 140379 |
| #14 | #1 OR #2 OR #3 OR #4 OR #11 | 292651 |
| #13 | perioperative care':ab,ti OR 'enhanced recovery after surgery':ab,ti OR 'enhanced recovery pathway':ab,ti OR 'fast track surgery':ab,ti OR 'fast track recovery':ab,ti OR 'eras':ab,ti OR 'fts':ab,ti | 20401 |
| #12 | 'endoscopic sinus surgery':ab,ti OR 'polypectomy':ab,ti OR 'sinus surgery':ab,ti OR 'fess':ab,ti OR 'ess':ab,ti | 33433 |
| #11 | 'sinusitis':ab,ti OR 'rhinosinusitis':ab,ti OR 'chronic rhinosinusitis':ab,ti OR 'chronic sinusitis':ab,ti OR 'nasosinusitis':ab,ti OR 'paranasal sinus disease':ab,ti OR 'nasal polyp':ab,ti OR 'sinus*':ab,ti OR 'crs':ab,ti | 273970 |
| #10 | 'fast track surgery'/exp | 117 |
| #9 | 'enhanced recovery after surgery'/exp | 3676 |
| #8 | 'perioperative care'/exp | 163226 |
| #7 | 'ear nose throat surgery'/exp | 112785 |
| #6 | 'sinus surgery'/exp | 51 |
| #5 | 'endoscopic sinus surgery'/exp | 6123 |
| #4 | 'chronic sinusitis'/exp | 5064 |
| #3 | 'chronic rhinosinusitis'/exp | 8625 |
| #2 | 'rhinosinusitis'/exp | 14219 |
| #1 | 'sinusitis'/exp | 53551 |

**Cochrane CENTRAL**

|  | Searches | Results |
| --- | --- | --- |
| #12 | #9 AND #10 AND #11 | 240 |
| #11 | #4 OR #5 OR #8 | 19837 |
| #10 | #2 OR #3 OR #7 | 11029 |
| #9 | #1 OR #6 | 15587 |
| #8 | (perioperative care):ti,ab,kw OR (enhanced recovery after surgey):ti,ab,kw OR (enhanced recovery pathway):ti,ab,kw OR (fast track surgery):ti,ab,kw OR (fast track recovery):ti,ab,kw OR (ERAS):ti,ab,kw OR (FTS):ti,ab,kw | 8575 |
| #7 | (endoscopic sinus surgery):ti,ab,kw OR (polypectomy):ti,ab,kw OR (sinus surgery):ti,ab,kw OR (FESS):ti,ab,kw OR (ESS):ti,ab,kw | 6668 |
| #6 | (sinusitis):ti,ab,kw OR (rhinosinusitis):ti,ab,kw OR (chronic rhinosinusitis):ti,ab,kw OR (chronic sinusitis):ti,ab,kw OR (nasosinusitis):ti,ab,kw OR (paranasal sinus disease):ti,ab,kw OR (nasal polyp):ti,ab,kw OR (sinus*):ti,ab,kw OR (CRS):ti,ab,kw | 15587 |
| #5 | MeSH descriptor: [Enhanced Recovery After Surgery] explode all trees | 131 |
| #4 | MeSH descriptor: [Perioperative Care] explode all trees | 13706 |
| #3 | MeSH descriptor: [Otorhinolaryngologic Surgical Procedures] explode all trees | 4507 |
| #2 | MeSH descriptor: [Nasal Surgical Procedures] explode all trees | 436 |
| #1 | MeSH descriptor: [Sinusitis] explode all trees | 1263 |

**Ovid**

|  | Searches | Results |
| --- | --- | --- |
| #4 | #1 AND #2 AND #3 | 12 |
| #3 | (Perioperative Care or enhanced recovery after surgey or enhanced recovery pathway or fast track surgery or fast track recovery or ERAS or FTS).ti,ab,kw. | 13480 |
| #2 | (Otorhinolaryngologic Surgical Procedures or nasal surgical procedures or endoscopic sinus surgery or polypectomy or sinus surgery or FESS or ESS).ti,ab,kw. | 20777 |
| #1 | (sinusitis or rhinosinusitis or chronic rhinosinusitis or chronic sinusitis or nasosinusitis or paranasal sinus disease or nasal polyp or sinus* or CRS).ti,ab,kw. | 197912 |

**CNKI**

|  | Searches | Results |
| --- | --- | --- |
| #4 | #1 AND #2 AND #3 | 29 |
| #3 | SU %= '加速康复' OR SU %= '快速康复' OR SU %= '快通道外科' OR SU %= '多模式围手术期护理' | 17313 |
| #2 | SU %= '内窥镜鼻窦手术' OR SU %= '内镜鼻窦手术' OR SU %= '鼻窦内镜手术' OR SU %= '鼻窦内窥镜手术' OR SU %= '鼻内镜手术' OR SU %= '鼻内镜术' | 14283 |
| #1 | SU %= '鼻窦炎' OR SU %= '慢性鼻窦炎' OR SU %= '慢性鼻鼻窦炎' | 19931 |

**Wanfang**

|  | Searches | Results |
| --- | --- | --- |
| #4 | #1 and #2 and #3 | 70 |
| #3 | 主题：（加速康复 or 快速康复 or 快通道外科 or 多模式围手术期护理） | 48420 |
| #2 | 主题：（内窥镜鼻窦手术 or 内镜鼻窦手术 or 鼻窦内镜手术 or 鼻窦内窥镜手术 or 鼻内镜手术 or 鼻内镜术） | 31003 |
| #1 | 主题：（鼻窦炎 or 慢性鼻窦炎 or 慢性鼻-鼻窦炎） | 27779 |

**CBM**

|  | Searches | Results |
| --- | --- | --- |
| #6 | (#5) AND (#4) AND (#3) | 29 |
| #5 | (#2) OR (#1) | 20389 |
| #4 | 加速康复[摘要:智能] OR "快速康复"[摘要:智能] OR "快通道外科"[摘要:智能] OR "多模式围手术期护理"[摘要:智能] | 17681 |
| #3 | 内窥镜鼻窦手术[摘要:智能] OR "内镜鼻窦手术"[摘要:智能] OR "鼻窦内镜手术"[摘要:智能] OR "鼻窦内窥镜手术"[摘要:智能] OR "鼻内镜手术"[摘要:智能] OR "鼻内镜术"[摘要:智能] | 8381 |
| #2 | 鼻窦炎[摘要:智能] OR "慢性鼻窦炎"[摘要:智能] OR "慢性鼻-鼻窦炎"[摘要:智能] | 17624 |
| #1 | "鼻窦炎"[不加权:扩展] | 14880 |

**VIP**

|  | Searches | Results |
| --- | --- | --- |
| #4 | 1 and 2 and 3 | 25 |
| #3 | 题名或关键词：加速康复+快速康复+快通道外科+多模式围手术期护理 | 16531 |
| #2 | 题名或关键词：内窥镜鼻窦手术+内镜鼻窦手术+鼻窦内镜手术+鼻窦内窥镜手术+鼻内镜手术+鼻内镜术 | 12657 |
| #1 | 题名或关键词：鼻窦炎+慢性鼻窦炎+慢性鼻-鼻窦炎 | 17183 |
