# Supplementary material for: The application of enhanced recovery after surgery (ERAS) in chronic rhinosinusitis patients undergoing endoscopic sinus surgery: A systematic review and meta-analysis
Source: PLoS One. 2023 Sep 21;18(9):e0291835. doi: 10.1371/journal.pone.0291835 (PMC10513253; doi:10.1371/journal.pone.0291835)
Supplement: S2 Appendix — (DOC) [file pone.0291835.s002.doc]

**S2 Appendix. Literature excluded after reading the full text and reasons**

**1) Not RCTs (n = 3)**

1. Gao YB, Wang CS, Wang GY, Cui X, Yang G, Lou HF, et al. Benefits of Enhanced Recovery After Surgery in Patients Undergoing Endoscopic Sinus Surgery. American Journal of Rhinology & Allergy. 2020;34(2):280-9.

2. Tian RX, Wang JW, Yang YJ, Liu LP, Sun YM, Tang NN, et al. Perioperative airway management based on enhanced recovery after surgery for improvement of preoperative pulmonary function in patients with aspirin intolerance triad. Chinese journal of otorhinolaryngology head and neck surgery. 2021;56(3):229-35.

3. Zhang XL. Application of accelerated rehabilitation surgical pathway in nasal endoscopic therapy. Zhejiang Journal of Traumatic Surgery. 2021;26(06):1086-7.

**2) Different participants (n = 2)**

1. Fan J. Preliminary application of the concept of accelerated rehabilitation surgery in perioperative nursing of functional endoscopic sinus surgery. Health Guide. 2019(28):154.

2. Zhang SJ, Wang L. Perioperative airway management based on enhanced recovery after surgery for improvement of preoperative pulmonary function in patients with aspirin intolerance triad. New Mom and New Born. 2022(5):173-4.

**3) ERAS elements＜6 (n = 2)**

1. Cen GF, Yang YS, Liu X, C., Liang J, X., Li C, J., Gao J, B. Study on the correlation of satisfaction with nursing services for patients undergoing nasal endoscopic surgery in rapid rehabilitation mode. China Medicine and Pharmacy. 2020;10(4).

2. Fu Y. Application effect analysis of accelerated rehabilitation surgical nursing in patients with chronic rhinosinusitis undergoing nasal endoscopic surgery. Diet Health. 2020(41):132.

**4) Different intervention (n = 2)**

1. Han XY, J MM. Application of accelerated rehabilitation combined with external treatment nursing of traditional Chinese medicine in endoscopic treatment of patients with chronic sinusitis. Healthmust-Readmagazine. 2022(15):30-1.

2. Jia Y, Ding N, Chen JN. Application of accelerated rehabilitation combined with external treatment of traditional Chinese medicine in the treatment of chronic sinusitis by nasal endoscopy. Hunan Journal of Traditional Chinese Medicine. 2021;37(9).

**5) Unavailable data (n = 2)**

1. Dong ZH. To analyze the application of accelerated rehabilitation nursing in patients with chronic sinusitis undergoing endoscopic surgery. Great health. 2020(12):129-30.

2. Pan Y, Ding L, Zhang J. Intervention effect of accelerated rehabilitation surgical nursing on patients with chronic sinusitis undergoing nasal endoscopic surgery. Health Guide. 2020(28):186-7.
