# Supplementary material for: The application of enhanced recovery after surgery (ERAS) in chronic rhinosinusitis patients undergoing endoscopic sinus surgery: A systematic review and meta-analysis
Source: PLoS One. 2023 Sep 21;18(9):e0291835. doi: 10.1371/journal.pone.0291835 (PMC10513253; doi:10.1371/journal.pone.0291835)
Supplement: S3 Appendix — (DOC) [file pone.0291835.s003.doc]

**S3 Appendix. Subgroup analysis of LOS for ERAS vs. SC.**


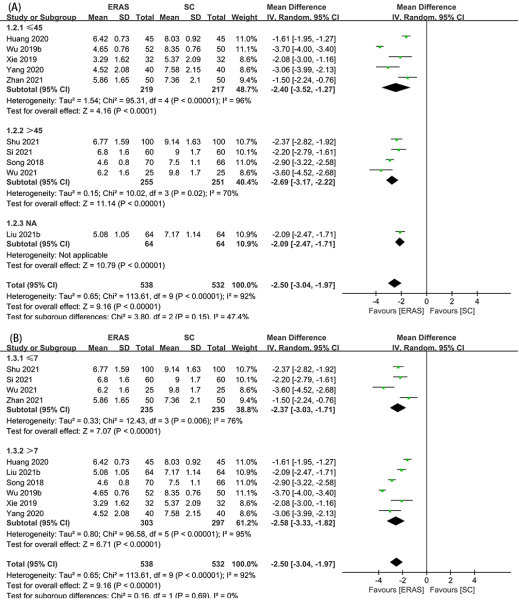


Additional file 3. (A)subgroup analysis of LOS by age. (B) subgroup analysis of LOS by ERAS elements.
