# Supplementary material for: The application of enhanced recovery after surgery (ERAS) in chronic rhinosinusitis patients undergoing endoscopic sinus surgery: A systematic review and meta-analysis
Source: PLoS One. 2023 Sep 21;18(9):e0291835. doi: 10.1371/journal.pone.0291835 (PMC10513253; doi:10.1371/journal.pone.0291835)
Supplement: S7 Appendix — (DOC) [file pone.0291835.s007.doc]

**S7 Appendix. Publication bias of LOS, overall complications, and VAS pain score.**


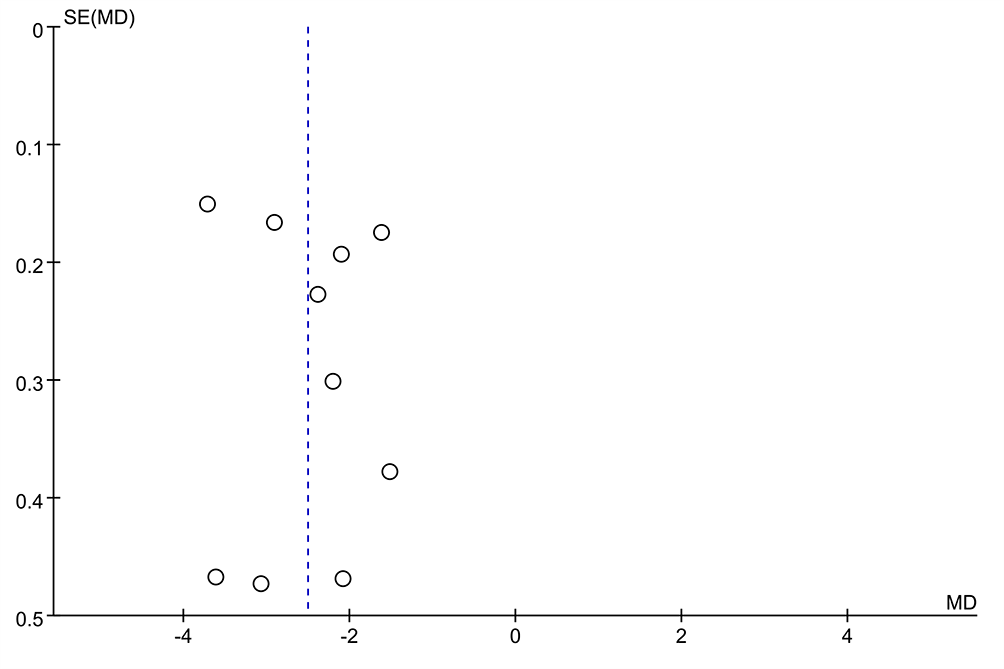


Figure1. Funnel plot test of LOS. (Begg’s test *p*=1.000; Egger’s test *p*=0.566)


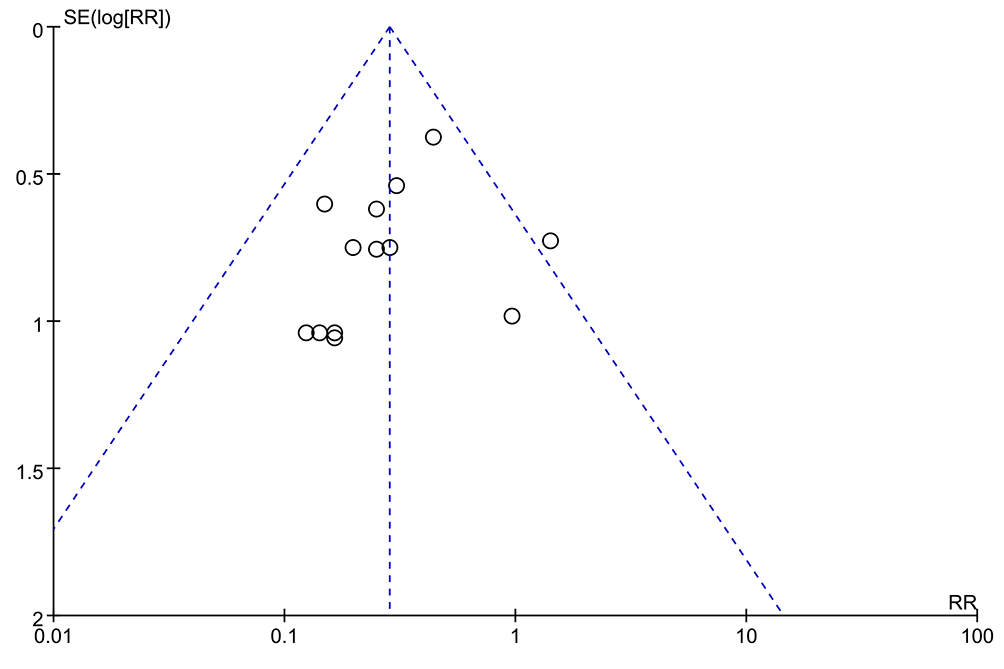


Figure2. Funnel plot test of overall complications. (Begg’s test *p*=0.200; B, Eegger’s test *p*= 0.241)


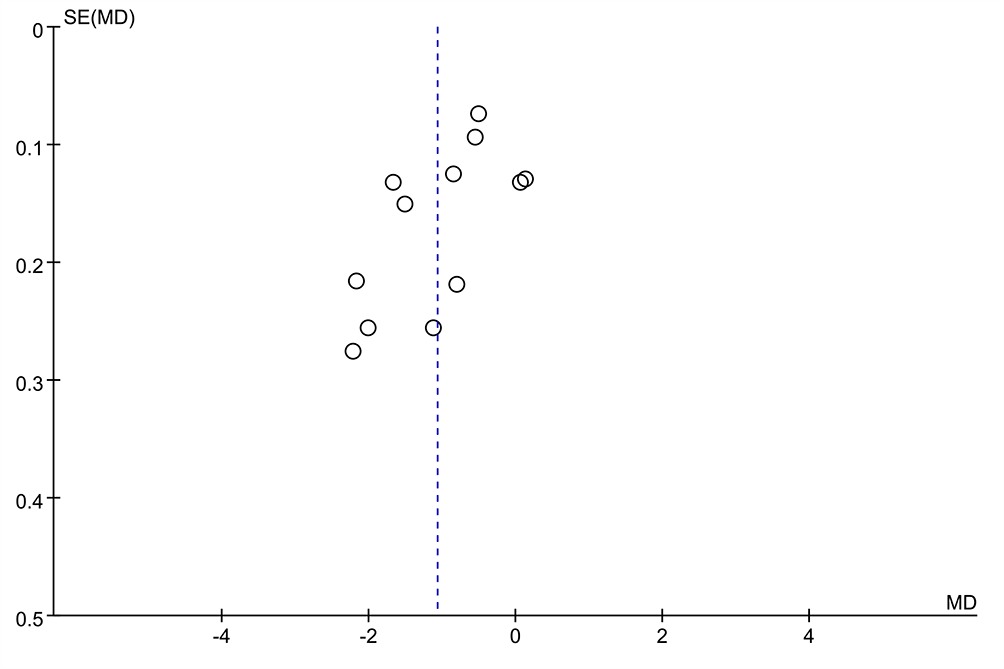


Figure3. Funnel plot test of VAS pain score. (Begg’s test *p*=0.150; Eegger’s test *p*= 0.057)
