# Supplementary material for: The application of enhanced recovery after surgery (ERAS) in chronic rhinosinusitis patients undergoing endoscopic sinus surgery: A systematic review and meta-analysis
Source: PLoS One. 2023 Sep 21;18(9):e0291835. doi: 10.1371/journal.pone.0291835 (PMC10513253; doi:10.1371/journal.pone.0291835)
Supplement: S8 Appendix — (DOC) [file pone.0291835.s008.doc]

**S8 Appendix. Sensitivity analysis.**


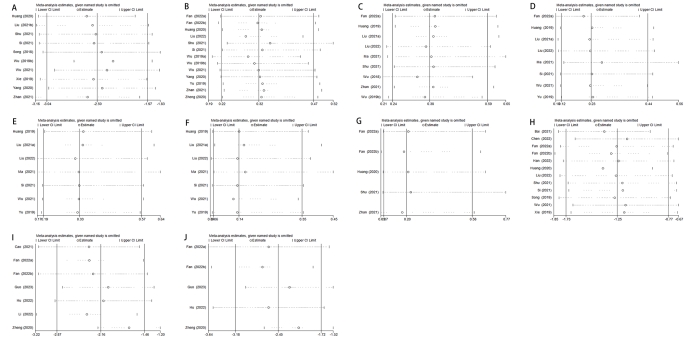
 Sensitivity analysis of ERAS vs SC: (A) LOS; (B)Overall complications; (C) PONV; (D) Facial edema; (E) Low back pain; (F) Urinary retention; (G) Haemorrhage; (H) VAS pain score; (I) Anxiety score; (J) Depression score.
