# Supplementary material for: The application of enhanced recovery after surgery (ERAS) in chronic rhinosinusitis patients undergoing endoscopic sinus surgery: A systematic review and meta-analysis
Source: PLoS One. 2023 Sep 21;18(9):e0291835. doi: 10.1371/journal.pone.0291835 (PMC10513253; doi:10.1371/journal.pone.0291835)
Supplement: S9 Appendix — (DOC) [file pone.0291835.s009.doc]

**S9 Appendix. Assessment of evidence quality for each outcome**

| **Quality assessment** | | | | | | | | | **No of patients** | | **Effect** | | **Quality** | **Importance** |  |
| --- | --- | --- | --- | --- | --- | --- | --- | --- | --- | --- | --- | --- | --- | --- | --- |
|  |
| **No of studies** | **Design** | | **Risk of bias** | | **Inconsistency** | **Indirectness** | **Imprecision** | **Other considerations** | **LOS**  **Control** | | **Relative**  **(95% CI)** | **Absolute** |  |
| **LOS (Better indicated by lower values)** | | | | | | | | | | | | | | |  |
| 10 | randomised trials | | serious^1^ | | very serious^2^ | no serious indirectness | no serious imprecision | none | 538 | 532 | - | MD 2.5 lower (3.04 to 1.97 lower) | ÅOOO  VERY LOW | CRITICAL |  |
| **Overall complications** | | | | | | | | | | | | | | |  |
| 13 | randomised trials | | serious^1^ | | no serious inconsistency | no serious indirectness | no serious imprecision | none | 34/637  (5.3%) | 120/637  (18.8%) | RR 0.28 (0.2 to 0.41) | 136 fewer per 1000 (from 111 fewer to 151 fewer) | ÅÅÅO  MODERATE | IMPORTANT |  |
| **PONV** | | | | | | | | | | | | | | |  |
| 9 | randomised trials | serious^1^ | | no serious inconsistency | | no serious indirectness | no serious imprecision | none | 23/463  (5%) | 73/463  (15.8%) | RR 0.33 (0.21 to 0.5) | 106 fewer per 1000 (from 79 fewer to 125 fewer) | ÅÅÅO  MODERATE | IMPORTANT |  |
| **Facial edema** | | | | | | | | | | | | | | |  |
| 8 | randomised trials | serious^1^ | | no serious inconsistency | | no serious indirectness | no serious imprecision | none | 10/344  (2.9%) | 53/344  (15.4%) | RR 0.2 (0.11 to 0.38) | 123 fewer per 1000 (from 96 fewer to 137 fewer) | ÅÅÅO  MODERATE | IMPORTANT |  |
| **Low back pain** | | | | | | | | | | | | | | |  |
| 7 | randomised trials | serious^1^ | | no serious inconsistency | | no serious indirectness | no serious imprecision | none | 15/284  (5.3%) | 53/284  (18.7%) | RR 0.28 (0.16 to 0.49) | 134 fewer per 1000 (from 95 fewer to 157 fewer) | ÅÅÅO  MODERATE | IMPORTANT |  |
| **Urinary retention** | | | | | | | | | | | | | | |  |
| 7 | randomised trials | serious^1^ | | no serious inconsistency | | no serious indirectness | no serious imprecision | none | 3/284  (1.1%) | 39/284  (13.7%) | RR 0.12 (0.05 to 0.3) | 121 fewer per 1000 (from 96 fewer to 130 fewer) | ÅÅÅO  MODERATE | IMPORTANT |  |
| **Haemorrhage** | | | | | | | | | | | | | | |  |
| 5 | randomised trials | serious^1^ | | no serious inconsistency | | no serious indirectness | no serious imprecision | none | 3/315  (1%) | 20/315  (6.3%) | RR 0.19 (0.07 to 0.55) | 51 fewer per 1000 (from 29 fewer to 59 fewer) | ÅÅÅO  MODERATE | IMPORTANT |  |
| **VAS score** | | | | | | | | | | | | | | |  |
| 12 | randomised trials | | serious^1^ | | very serious^2^ | no serious indirectness | no serious imprecision | none | 639 | 639 | - | MD 1.07 lower (1.46 to 0.67 lower) | ÅOOO  VERY LOW | IMPORTANT |  |
| **Anxiety score** | | | | | | | | | | | | | | |  |
| 7 | randomised trials | | serious^1^ | | very serious^2^ | no serious indirectness | no serious imprecision | none | 298 | 296 | - | SMD 2.13 lower (2.83 to 1.44 lower) | ÅOOO  VERY LOW | IMPORTANT |  |
| **Depression score** | | | | | | | | | | | | | | |  |
| 5 | randomised trials | | serious^1^ | | very serious^2^ | no serious indirectness | no serious imprecision | none | 214 | 214 | - | SMD 2.42 lower (3.13 to 1.71 lower) | ÅOOO  VERY LOW | IMPORTANT |  |

^1^ Risk of bias was downgraded because most of the included RCTs were at moderate risk of bias due to incomplete data.

^2^ There were large differences in point estimates between studies, small overlap of confidence intervals, or large heterogeneity.

Abbreviations: ERAS, Enhanced Recovery After Surgery; SC, standard care; CI, confidence interval; MD, mean difference; SMD; standardized mean difference; RR, risk ratio.
