# Supplementary material for: The application of enhanced recovery after surgery (ERAS) in chronic rhinosinusitis patients undergoing endoscopic sinus surgery: A systematic review and meta-analysis
Source: PLoS One. 2023 Sep 21;18(9):e0291835. doi: 10.1371/journal.pone.0291835 (PMC10513253; doi:10.1371/journal.pone.0291835)
Supplement: Peer review history

# Peer review: Decision Letter 0

**DOI:** 10.1371/journal.pone.0291835.r001

**Authors:** Amirmohammad Khalaji

29 Aug 2023

PONE-D-23-24761The application of enhanced recovery after surgery (ERAS) in chronic rhinosinusitis patients undergoing endoscopic sinus surgery: a systematic review and meta-analysisPLOS ONE

Dear Dr. Wu,

Thank you for submitting your manuscript to PLOS ONE. After careful consideration, we feel that it has merit but does not fully meet PLOS ONE’s publication criteria as it currently stands. Therefore, we invite you to submit a revised version of the manuscript that addresses the points raised during the review process.

==============================

ACADEMIC EDITOR: We will reconsider your paper for publication after revising your manuscript based on comments from reviewers 1 and 2.

==============================

Please submit your revised manuscript by Oct 13 2023 11:59PM. If you will need more time than this to complete your revisions, please reply to this message or contact the journal office at plosone@plos.org. When you're ready to submit your revision, log on to https://www.editorialmanager.com/pone/ and select the 'Submissions Needing Revision' folder to locate your manuscript file.

Please include the following items when submitting your revised manuscript:

- A rebuttal letter that responds to each point raised by the academic editor and reviewer(s). You should upload this letter as a separate file labeled 'Response to Reviewers'.
- A marked-up copy of your manuscript that highlights changes made to the original version. You should upload this as a separate file labeled 'Revised Manuscript with Track Changes'.
- An unmarked version of your revised paper without tracked changes. You should upload this as a separate file labeled 'Manuscript'.

If you would like to make changes to your financial disclosure, please include your updated statement in your cover letter. Guidelines for resubmitting your figure files are available below the reviewer comments at the end of this letter.

If applicable, we recommend that you deposit your laboratory protocols in protocols.io to enhance the reproducibility of your results. Protocols.io assigns your protocol its own identifier (DOI) so that it can be cited independently in the future. For instructions see: https://journals.plos.org/plosone/s/submission-guidelines#loc-laboratory-protocols. Additionally, PLOS ONE offers an option for publishing peer-reviewed Lab Protocol articles, which describe protocols hosted on protocols.io. Read more information on sharing protocols at https://plos.org/protocols?utm_medium=editorial-email&utm_source=authorletters&utm_campaign=protocols.

We look forward to receiving your revised manuscript.

Kind regards,

Amirmohammad Khalaji

Academic Editor

PLOS ONE

Journal Requirements:

When submitting your revision, we need you to address these additional requirements.

1. Please ensure that your manuscript meets PLOS ONE's style requirements, including those for file naming. The PLOS ONE style templates can be found at 

https://journals.plos.org/plosone/s/file?id=wjVg/PLOSOne_formatting_sample_main_body.pdf and 

https://journals.plos.org/plosone/s/file?id=ba62/PLOSOne_formatting_sample_title_authors_affiliations.pdf

2. Thank you for submitting the above manuscript to PLOS ONE. During our internal evaluation of the manuscript, we found significant text overlap between your submission and previous work in the [introduction, conclusion, etc.].

We would like to make you aware that copying extracts from previous publications, especially outside the methods section, word-for-word is unacceptable. In addition, the reproduction of text from published reports has implications for the copyright that may apply to the publications.

Please revise the manuscript to rephrase the duplicated text, cite your sources, and provide details as to how the current manuscript advances on previous work. Please note that further consideration is dependent on the submission of a manuscript that addresses these concerns about the overlap in text with published work.

[If the overlap is with the authors’ own works: Moreover, upon submission, authors must confirm that the manuscript, or any related manuscript, is not currently under consideration or accepted elsewhere. If related work has been submitted to PLOS ONE or elsewhere, authors must include a copy with the submitted article. Reviewers will be asked to comment on the overlap between related submissions (http://journals.plos.org/plosone/s/submission-guidelines#loc-related-manuscripts).]

We will carefully review your manuscript upon resubmission and further consideration of the manuscript is dependent on the text overlap being addressed in full. Please ensure that your revision is thorough as failure to address the concerns to our satisfaction may result in your submission not being considered further.

3. Thank you for stating the following financial disclosure: 

"Hui Li

Natural Science Foundation of China (81674037)

 the Sichuan Science and Technology Support Program (2022YFS0421)

Hui Li: Methodology and Writing – review & editing"

Please state what role the funders took in the study.  If the funders had no role, please state: "The funders had no role in study design, data collection and analysis, decision to publish, or preparation of the manuscript." 

If this statement is not correct you must amend it as needed. 

Please include this amended Role of Funder statement in your cover letter; we will change the online submission form on your behalf.

4. Please include captions for your Supporting Information files at the end of your manuscript, and update any in-text citations to match accordingly. Please see our Supporting Information guidelines for more information: http://journals.plos.org/plosone/s/supporting-information. 

[Note: HTML markup is below. Please do not edit.]

Reviewers' comments:

Reviewer's Responses to Questions

**Comments to the Author**

1. Is the manuscript technically sound, and do the data support the conclusions?

The manuscript must describe a technically sound piece of scientific research with data that supports the conclusions. Experiments must have been conducted rigorously, with appropriate controls, replication, and sample sizes. The conclusions must be drawn appropriately based on the data presented. 

Reviewer #1: Yes

Reviewer #2: Yes

**********

2. Has the statistical analysis been performed appropriately and rigorously? 

Reviewer #1: Yes

Reviewer #2: Yes

**********

3. Have the authors made all data underlying the findings in their manuscript fully available?

The PLOS Data policy requires authors to make all data underlying the findings described in their manuscript fully available without restriction, with rare exception (please refer to the Data Availability Statement in the manuscript PDF file). The data should be provided as part of the manuscript or its supporting information, or deposited to a public repository. For example, in addition to summary statistics, the data points behind means, medians and variance measures should be available. If there are restrictions on publicly sharing data—e.g. participant privacy or use of data from a third party—those must be specified.

Reviewer #1: Yes

Reviewer #2: Yes

**********

4. Is the manuscript presented in an intelligible fashion and written in standard English?

PLOS ONE does not copyedit accepted manuscripts, so the language in submitted articles must be clear, correct, and unambiguous. Any typographical or grammatical errors should be corrected at revision, so please note any specific errors here.

Reviewer #1: Yes

Reviewer #2: Yes

**********

5. Review Comments to the Author

Please use the space provided to explain your answers to the questions above. You may also include additional comments for the author, including concerns about dual publication, research ethics, or publication ethics. (Please upload your review as an attachment if it exceeds 20,000 characters)

Reviewer #1: Observations

The study was a systhematic evaluation and meta-analysis which aimed at assessing the efficacy and safety of the Enhanced Recovery after Surgery (ERAS). This involved patients with Chronic sinus disease, who had undergone Endoscopic Sinus Surgery (ESS) as a treatment option. Risk of bias and quality of evidence were also assessed. Study period was from inception to Feb, 2023.

Overall, study pooled data from 28 RCTs which involved 2636 patients, which was quite robust and representative. Research was well-thought out and executed, being capable of contributing to the body of knowledge on the subject research. However, I have the following observations,

1. Title: The title is appropriate and reflects the study (systhematic review and meta-analysis)

2. Methods: In line 138, the statement, then we consulted experts to determine our search strategy, is not clear. Kindly clarify.

-Also, in lines 168 to 170, how did we come about the use of <45 year to explore potential sources of heterogeneity? Was this choice random?

3. Results: The abstract as well as the introductory aspects of the manuscript described the study period as being from inception to Feb, 2023. However, the RCTs utilized were published between 2018 and 2023 (line 193-194). Could be harmonize these discrepancies to have uniformity?

-Is there a possibility of a bias in the study, given the variation in the characteristics of the patients? CRSwNP + CRSsNP patients (in 24 studies) and CRSwNP in the remaining 4 studies.

-Also, if the primary outcome from the inclusion criteria is length of stay (LOS) was only reported in 10/28 RCTs reviewed, is the finding representative?

4. Discussion: The prevalence of individuals that were vaccinated with HPV vaccine was reported as being only 2. This was mentioned in the discussion and a correlation with HPV status and possible recurrence would have been helpful.

-Absence of contributory factors to HPV infection such as smoking and sexual activity were reported as limitations due to the retrospective nature of the study.

5. Conclusion: I agree with the finding that more larger studies would be required to be carried out to give clearer insights into challenges posed by heterogeneity and risk of bias.

6. I found the title, study design, statistics, discussion, figures and tables, referencing quite satisfactory.

Reviewer #2: Wu et al. conducted an interesting study and evaluated the effect of ERAS on CRS patients undergoing ESS. The methods are appropriate and well-designed. Minor issues should be addressed:

Language: The full manuscript should be revised, Some the examples are as follows:

1. Line 85: “traction” doesn’t fit the sentence and should be substituted by “attention” or other synonyms.

2. Line 102-103: “evidence-based evidence” should be replaced by a better phrase.

3. Line 126: The first item should change to “Studies that are not RCTs”

4. Line 148: “mainly” should be removed.

Method:

1. Use the updated PRISMA flowchart (2020) for Figure 1

2. Line 122: Like the pain score, it should be explained that quality of life, anxiety score, and depressive score are assessed based on what scales.

3. Line 166: Need references for Hozo et al. and Luo et al.

4. Line 169-170: Use brackets inside parentheses to create a double enclosure in the text.

5. Line 343: “As it is well-known”

6. Line 371-372: Use a comma after both “management”s

Results:

1. Redesign Table 2 so it is easier to read.

2. Expand the qualitative synthesis so it would be more comprehensive of the 28 included studies and important insights that were not used in the meta-analysis.

Discussion:

1. Provide more explanation in the conclusion section and suggest how future studies should be performed so they may extend and confirm your findings.

**********

6. PLOS authors have the option to publish the peer review history of their article (what does this mean?). If published, this will include your full peer review and any attached files.

If you choose “no”, your identity will remain anonymous but your review may still be made public.

**Do you want your identity to be public for this peer review?** For information about this choice, including consent withdrawal, please see our Privacy Policy.

Reviewer #1: **Yes: **Stephen Adebola

Reviewer #2: No

**********

[NOTE: If reviewer comments were submitted as an attachment file, they will be attached to this email and accessible via the submission site. Please log into your account, locate the manuscript record, and check for the action link "View Attachments". If this link does not appear, there are no attachment files.]

While revising your submission, please upload your figure files to the Preflight Analysis and Conversion Engine (PACE) digital diagnostic tool, https://pacev2.apexcovantage.com/. PACE helps ensure that figures meet PLOS requirements. To use PACE, you must first register as a user. Registration is free. Then, login and navigate to the UPLOAD tab, where you will find detailed instructions on how to use the tool. If you encounter any issues or have any questions when using PACE, please email PLOS at figures@plos.org. Please note that Supporting Information files do not need this step.

# Author comment: Author response to Decision Letter 0

**DOI:** 10.1371/journal.pone.0291835.r002

4 Sep 2023

Response to Academic Editor and Reviewers

Thank you for giving us the opportunity to revise our manuscript entitled “The application of enhanced recovery after surgery (ERAS) in chronic rhinosinusitis patients undergoing endoscopic sinus surgery: a systematic review and meta-analysis”.

We sincerely appreciate the editors and reviewers for the thorough and knowledgeable review, which helped us optimize the manuscript and provided significant guidance for our future research. We did our best to respond to the editor's and reviewer's comments and tracked our changes in the revised manuscript. We hope that our response and the revised manuscript are satisfactory and sincerely hope that the revised manuscript will be accepted for publication in PLOS ONE.

Since our manuscript has been revised for text overlap and language touch-ups, we have highlighted the changes in this section in red. In the revised manuscript for editorial and reviewer comments, we have made changes in red and highlighted them with a yellow marker.

Journal Requirements:

When submitting your revision, we need you to address these additional requirements.

1. Please ensure that your manuscript meets PLOS ONE's style requirements, including those for file naming. The PLOS ONE style templates can be found at

https://journals.plos.org/plosone/s/file?id=wjVg/PLOSOne_formatting_sample_main_body.pdf and

https://journals.plos.org/plosone/s/file?id=ba62/PLOSOne_formatting_sample_title_authors_affiliations.pdf

Authors’ Response: 

Thank you for your comments. We have reviewed the PLOS ONE template and made the required changes to ensure that our manuscript meets PLOS ONE's style requirements. At the same time, we have also checked our file names.

2. Thank you for submitting the above manuscript to PLOS ONE. During our internal evaluation of the manuscript, we found significant text overlap between your submission and previous work in the [introduction, conclusion, etc.].

We would like to make you aware that copying extracts from previous publications, especially outside the methods section, word-for-word is unacceptable. In addition, the reproduction of text from published reports has implications for the copyright that may apply to the publications.

Please revise the manuscript to rephrase the duplicated text, cite your sources, and provide details as to how the current manuscript advances on previous work. Please note that further consideration is dependent on the submission of a manuscript that addresses these concerns about the overlap in text with published work.

[If the overlap is with the authors’ own works: Moreover, upon submission, authors must confirm that the manuscript, or any related manuscript, is not currently under consideration or accepted elsewhere. If related work has been submitted to PLOS ONE or elsewhere, authors must include a copy with the submitted article. Reviewers will be asked to comment on the overlap between related submissions (http://journals.plos.org/plosone/s/submission-guidelines#loc-related-manuscripts).]

We will carefully review your manuscript upon resubmission and further consideration of the manuscript is dependent on the text overlap being addressed in full. Please ensure that your revision is thorough as failure to address the concerns to our satisfaction may result in your submission not being considered further.

Authors’ Response: 

Thank you for your rigorous review and careful consideration. We are very apologetic about the apparent overlapping of text in the manuscript, we are aware of this serious error and have carefully scrutinized every part of the manuscript and revised it to the best of our ability. We have used professional plagiarism detection software to check the duplication rate of our revised manuscript and we hope that the revised result will satisfy you. If you have any other suggestions, we will be happy to hear them and take them on board. For the modifications, we have used red font for the changes.

3. Thank you for stating the following financial disclosure:

"Hui Li

Natural Science Foundation of China (81674037)

 the Sichuan Science and Technology Support Program (2022YFS0421)

Hui Li: Methodology and Writing – review & editing"

Please state what role the funders took in the study. If the funders had no role, please state: "The funders had no role in study design, data collection and analysis, decision to publish, or preparation of the manuscript."

If this statement is not correct you must amend it as needed.

Please include this amended Role of Funder statement in your cover letter; we will change the online submission form on your behalf.

Authors’ Response: 

Thank you for your comments. We have added a description of the funder's role to the Funds section as requested.

Revised text: (Page 31, lines 566-567)

4. Please include captions for your Supporting Information files at the end of your manuscript, and update any in-text citations to match accordingly. Please see our Supporting Information guidelines for more information: http://journals.plos.org/plosone/s/supporting-information.

Authors’ Response: 

Thank you for your comments. We apologize for our oversight. We have added the captions of Supporting Information files at the end of my manuscript (before the references section). Meanwhile, we have updated the citations after double-checking the full text. Notably, we removed the citation after realizing that Xu et al.'s study (DOI: 10.1155/2023/9893414) status has now been updated to a retracted article. We hope that our revisions will satisfy you.

Responds to the reviewer’s comments:

Reviewer #1:

1. Title: The title is appropriate and reflects the study (systhematic review and meta-analysis)

Authors’ response: 

Thank you very much for your approval of our design of the title.

2. Methods: In line 138, the statement, then we consulted experts to determine our search strategy, is not clear. Kindly clarify.

Authors’ response:

Thank you for your comments. We apologize for the unclear presentation. We will explain and revise the language here. Our work is a part of the project funded by the Natural Science Foundation of China (81674037) and the Sichuan Science and Technology Support Program (2022YFS0421). Therefore the search strategy for this work was guided by the two funding project teams that included information specialists. we intended to convey whether, after consulting with information experts in the relevant fields, the experts were satisfied with the search strategy we developed and whether they could suggest refinements to our search strategy that would complement the relevant potential research as much as possible. We have modified the description of this section of the search strategy to improve the clarity of the content. 

Revised text: (Page 7, lines 147-149)

3.-Also, in lines 168 to 170, how did we come about the use of <45 year to explore potential sources of heterogeneity? Was this choice random?

Authors’ response:

Thank you for your rigorous consideration. We will clarify this point here by explaining the subgroup basis with a 45-year-old limit. Studies have found that with age, the nasal mucosa shows anatomic and functional changes associated with aging. Compared with young people, decreased ciliary function of the nasal mucosa and reduced nasal mucus secretion in older individuals result in thick and stagnant nasal mucus. In addition, with the increase of microbial colonization and atrophy and thinning of the nasal mucosa, the epithelial barrier is more severely damaged than that of young people, which may lead to the continuous development of the disease. Furthermore, it has been found that immune senescence may reduce the rate of wound healing. (DOI: 10.1177/0194599817691258 and DOI: 10.1007/s11882-019-0880-4) Therefore, we performed subgroup analyses according to age to investigate whether age may be a factor influencing the heterogeneity of the study results.

At present, most researchers and the public generally use 45 years of age as the dividing node between the middle-aged and the young, and 65 years of age as the dividing node between the middle-aged and the elder. Through consulting relevant literature, it is found that the definition of this age range has been involved in studies in the United States (DOI: 10.1016/j.msard.2020.102334), Norway (DOI: 10.1093/ije/dyr205), Sweden (DOI： 10.1111/cid.12753), the United Kingdom (DOI: 10.1093/rheumatology/key156), and China (DOI: 10.1007/s11255-023-03683-1) among other countries. Furthermore, in our included studies, we observed that the lowest mean age among all patients was 34.5 ± 3.8 years old, and the highest mean age was 59.90 ± 5.24 years old, both of which moved up and down the 45-year node with middle-aged and young adults as the dividing line. Therefore, in the subgroup analysis by age, we chose to divide the subgroups by the age threshold of 45 years for middle-aged and young adults.

4. Results: The abstract as well as the introductory aspects of the manuscript described the study period as being from inception to Feb, 2023. However, the RCTs utilized were published between 2018 and 2023 (line 193-194). Could be harmonize these discrepancies to have uniformity?

Authors’ response: 

Thank you for your valuable comments. We apologize that we may not have expressed ourselves clearly here, and we will explain and correct it here. We conducted a comprehensive search of all relevant RCTs published up to February 2023 across nine databases, and after reviewing them fully against eligibility criteria, a total of 28 articles were included in this study. Upon review, we found that the literature included in this study was published between 2018 and 2023.

ERAS is an emerging field of research that has only begun to be applied in CRS patients in recent years, and the number of scholars' studies in this area has only gradually increased in the past few years. After our rigorous review, we did not find any relevant studies published before 2018, which may be related to the fact that ERAS is an emerging care modality in CRS. The description of the year of publication in our study may have caused errors in the understanding of scholars; therefore, we removed the description of the year of inclusion in the study characteristics section of the results.

Revised text: (Page 10, line 205)

5. -Is there a possibility of a bias in the study, given the variation in the characteristics of the patients? CRSwNP + CRSsNP patients (in 24 studies) and CRSwNP in the remaining 4 studies.

Authors’ response: 

Thank you for your important advice. Your concern is correct. Since there were only four studies of CRSwNP in the included studies, we were unable to perform subgroup analyses by disease classification of patients to explore whether different disease types might be potential sources of heterogeneity. We will add a note on the possibility that disease categorization of CRS patients may have contributed to the bias of the study results in the Limitations section of the Discussion. 

Revised text: (Page 30, lines 536-538)

6.-Also, if the primary outcome from the inclusion criteria is length of stay (LOS) was only reported in 10/28 RCTs reviewed, is the finding representative?

Authors’ response: 

Thank you for your comments. I will answer the question about the choice of primary outcome indicators here. We believe that the choice of length of stay (LOS) as the primary outcome indicator is of some degree of value significance. Unlike usual care, the Enhanced Recovery After Surgery (ERAS) program is a perioperative, evidence-based model of recovery care that involves a multidisciplinary team working together to combine multiple integrated care modalities. The ERAS protocol aims to significantly improve patient recovery rates, reduce complications, and ultimately shorten patient LOS through a care pathway that combines surgery with perioperative evidence-based medical interventions. If perioperative patients experience pain problems, mental health issues, and postoperative complications, this can delay the recovery process and prolong the patient's LOS, thereby increasing the cost of hospitalization. It can be seen that the length of hospital stay is a key indicator to measure the degree of recovery from various diseases, and is a direct factor affecting the cost of hospitalization.

In previous meta-analyses on hepatobiliary (DOI: 10.1007/s11605-019-04499-0), gynecological (DOI: 10.3389/fmed.2021.694385), gastric (DOI: 10.1186/s12957-018-1309-6), urology surgery (DOI: 10.3389/fonc.2020.541390), and other diseases, significant and meaningful reductions in the LOS of patients after the implementation of ERAS protocols have been observed, demonstrating that ERAS protocols are positively beneficial in reducing the LOS.

Therefore, we set the LOS, which represents the degree of recovery of patients in the perioperative period, as the primary outcome indicator of this study. At the same time, we set other factors such as complications, pain score, mental health problems, etc. that may affect the LOS as secondary outcome indicators. The reason for this is that in addition to the primary outcome indicator of LOS, we also wanted to explore the effectiveness of ERAS on the recovery of perioperative complications, pain, anxiety, and depression, as well as the quality of life and hospitalization costs in CRS patients. Therefore, in addition to including relevant literature containing the outcome metric of length of hospitalization, we also included relevant literature containing the secondary outcomes in this study to more comprehensively analyze the efficacy and safety of the ERAS protocol applied to the perioperative period in CRS patients. By analyzing these secondary outcome indicators, to a certain extent, it can also indirectly reflect the degree of recovery from the side, thus affecting the LOS.

Although length of hospitalization is a key endpoint factor in testing the effectiveness of ERAS protocols, however, only 10 studies in this study analyzed LOS as an outcome indicator. This may be attributed to the different focus on the degree of disease recovery that the studies wanted to examine, or the lack of a well-developed study design, or the fact that there were fewer RCTs of relevant studies. This necessitates that scholars should conduct more high-quality RCTs in the future that include LOS, which is a representative outcome reflecting the degree of recovery from ERAS, as a study outcome, in order to provide a more comprehensive analysis of LOS after perioperative implementation of ERAS in CRS patients. In general, the analysis of LOS and other secondary outcomes in this study obtained beneficial results, that is, directly and indirectly, reflected that the implementation of ERAS protocol during the ESS perioperative period in CRS patients is meaningful to shorten the LOS in patients.

4. Discussion: The prevalence of individuals that were vaccinated with HPV vaccine was reported as being only 2. This was mentioned in the discussion and a correlation with HPV status and possible recurrence would have been helpful.

-Absence of contributory factors to HPV infection such as smoking and sexual activity were reported as limitations due to the retrospective nature of the study.

Authors’ response: 

Thank you very much for your advice. However, after a careful review of our study, we did not find any description of HPV in the discussion section or the full text of this study. We would be happy if you could help point out this part of the manuscript so that we can correct it.

5. Conclusion: I agree with the finding that more larger studies would be required to be carried out to give clearer insights into challenges posed by heterogeneity and risk of bias.

Authors’ response: 

Thank you for your comments. Thank you for recognizing our conclusions.

6. I found the title, study design, statistics, discussion, figures and tables, referencing quite satisfactory.

Authors’ response: 

Thank you very much for your positive comments on our study design and content.

Reviewer #2:

Wu et al. conducted an interesting study and evaluated the effect of ERAS on CRS patients undergoing ESS. The methods are appropriate and well-designed. Minor issues should be addressed:

Language: The full manuscript should be revised, Some the examples are as follows:

Authors’ response: 

Thank you for your comments. In order to keep our manuscript grammatically and linguistically correct, we looked for a friend who speaks English as a dominant language to ask for help in polishing it. We have corrected the linguistic errors in the manuscript and marked the changes in red. We hope that you will be satisfied with the grammatical and linguistic presentation of the revised manuscript.

1. Line 85: “traction” doesn’t fit the sentence and should be substituted by “attention” or other synonyms.

Authors’ response:

Thank you very much for your suggestion. We apologize for the error in our expression. We have replaced “traction” with “attention” in our manuscript.

Revised text: (Page 4, line 86)

2. Line 102-103: “evidence-based evidence” should be replaced by a better phrase.

Authors’ response:

Thank you for your suggestion. We have replaced the phrase "evidence-based evidence" with "Evidence-based support" in the manuscript, which we hope will satisfy you.

Revised text: (Page 5, line 104)

3. Line 126: The first item should change to “Studies that are not RCTs”

Authors’ response: 

Thank you very much for your comments. We are very sorry for our negligence. We have changed it to "Studies that are not RCTs" in the manuscript.

Revised text: (Page 7, line 135)

4. Line 148: “mainly” should be removed.

Authors’ response:

Thank you very much for your careful observations and suggestions. We apologize for our misrepresentation. We have removed “mainly” from the manuscript.

Revised text: (Page 8, line 158)

Method:

1. Use the updated PRISMA flowchart (2020) for Figure 1

Authors’ response: 

Thank you very much for your suggestion. We have replaced Figure 1 with the 2020 updated PRISMA flowchart.

2. Line 122: Like the pain score, it should be explained that quality of life, anxiety score, and depressive score are assessed based on what scales.

Authors’ response: 

Thank you for your thoughtful and rigorous consideration. We have added the scales for anxiety, depression, and quality of life to the eligibility criteria in the Materials and Methods section. In addition to this, we have added what scales were used in the different studies individually in the corresponding section of the results.

Revised text: (Page 6, lines 125-131)

 (Page 20, lines 285-289)

 (Page 21, lines 326-331)

3. Line 166: Need references for Hozo et al. and Luo et al.

Authors’ response:

Thank you for your careful observations and suggestions. We apologize for our carelessness. We have added references citing Hozo et al. and Luo et al. in the corresponding sections of the manuscript.

Revised text: (Page 9, line 177)

4. Line 169-170: Use brackets inside parentheses to create a double enclosure in the text.

Authors’ response:

Thank you very much for your suggestion. It was important for us to change the correct formatting of the manuscript. We have changed the use of parentheses in the Meta-analysis in the Methods section, and I hope that the changed format is correct and satisfactory to you.

Revised text: (Page 9, lines 180-182)

5. Line 343: “As it is well-known”

Authors’ response:

Thank you very much for your suggestion. Thank you very much for your suggestion. We have decided to replace "As it is well known" with "It is well-known that" in order to make the statement more fluent, and we hope that you will be satisfied with the changed statement.

Revised text: (Page 25, line 410)

6. Line 371-372: Use a comma after both “management”s

Authors’ response:

Thank you very much for your suggestion. We have taken your comments on board and have added commas after both "management" to make the grammar correct.

Revised text: (Page 26, lines 438-439)

Results:

1. Redesign Table 2 so it is easier to read.

Authors’ response:

Thank you very much for your proposal. We will revise Table 2 in the study characteristics in the results section to make it clearer.

Revised text: (Page 13-17, line 225)

2. Expand the qualitative synthesis so it would be more comprehensive of the 28 included studies and important insights that were not used in the meta-analysis.

Authors’ response:

Thank you for your comments. We have carefully considered your comments and put forward our considerations. Here, we explain the selection of indicators for qualitative analysis in our manuscript. In addition, after careful consideration of your suggestions, we have decided to expand the descriptive analysis of quality of life and hospitalization expenses in our manuscript and to add additional descriptions of other outcomes that were not included in this study. We hope that the revised results will be satisfactory to you.

Meta-analysis is a statistical strategy for research that quantitatively combines data using the same or similar research methods (DOI: 10.1111/j.1365-2702.2012.04122.x). At present, most systematic reviews and meta-analyses include both quantitative synthesis and qualitative synthesis. As scholars tend to analyze multiple outcome indicators of interest, it is inevitable that there may be problems such as high heterogeneity, different evaluation methods, or limited included studies for some outcome indicators, resulting in an inability to quantitatively merge the data to visualize the results of the comprehensive data. At this time, scholars usually choose to report results by descriptive analysis of outcome indicators that cannot be synthesized quantitatively (DOI: 10.1136/bmj-2021-068302; DOI: 10.3389/fmolb.2022.843810; DOI： 10.1371/journal.pone.0190892).

In this study, we included two outcome indicators, quality of life and hospitalization costs, in the qualitative analysis. The reason for this is that, although QOL was included in a total of 10 studies, the evaluation criteria were inversely related to the severity of CRS disease in eight of them and positively related to the severity of CRS disease in two of them. We performed descriptive analyses of the QOL outcome indicators as the 10 studies had different evaluation criteria and data could not be combined.

As hospitalization expenses were the outcome indicator of interest to us, but only one study reported hospitalization expenses, there were not enough studies to allow for a quantitative synthesis of this outcome indicator, so we subjected hospitalization expenses to a descriptive analysis.

Revised text: (Page 21-23, lines 311-377)

Discussion:

1. Provide more explanation in the conclusion section and suggest how future studies should be performed so they may extend and confirm your findings.

Authors’ response:

Thank you for your rigorous consideration. Based on your suggestions, we have added more explanations and a vision for the future in the discussion section, which we hope will satisfy you.

Revised text: (Page 28-29, lines 493-527)

Attachment Submitted filename: Response to Reviewers.doc Click here for additional data file.

# Peer review: Decision Letter 1

**DOI:** 10.1371/journal.pone.0291835.r003

**Authors:** Amirmohammad Khalaji

6 Sep 2023

The application of enhanced recovery after surgery (ERAS) in chronic rhinosinusitis patients undergoing endoscopic sinus surgery: a systematic review and meta-analysis

PONE-D-23-24761R1

Dear Dr. Wu,

We’re pleased to inform you that your manuscript has been judged scientifically suitable for publication and will be formally accepted for publication once it meets all outstanding technical requirements.

Within one week, you’ll receive an e-mail detailing the required amendments. When these have been addressed, you’ll receive a formal acceptance letter and your manuscript will be scheduled for publication.

An invoice for payment will follow shortly after the formal acceptance. To ensure an efficient process, please log into Editorial Manager at http://www.editorialmanager.com/pone/, click the 'Update My Information' link at the top of the page, and double check that your user information is up-to-date. If you have any billing related questions, please contact our Author Billing department directly at authorbilling@plos.org.

If your institution or institutions have a press office, please notify them about your upcoming paper to help maximize its impact. If they’ll be preparing press materials, please inform our press team as soon as possible -- no later than 48 hours after receiving the formal acceptance. Your manuscript will remain under strict press embargo until 2 pm Eastern Time on the date of publication. For more information, please contact onepress@plos.org.

Kind regards,

Amirmohammad Khalaji

Academic Editor

PLOS ONE

Additional Editor Comments (optional):

Reviewers' comments:

Reviewer's Responses to Questions

**Comments to the Author**

1. If the authors have adequately addressed your comments raised in a previous round of review and you feel that this manuscript is now acceptable for publication, you may indicate that here to bypass the “Comments to the Author” section, enter your conflict of interest statement in the “Confidential to Editor” section, and submit your "Accept" recommendation.

Reviewer #2: All comments have been addressed

**********

2. Is the manuscript technically sound, and do the data support the conclusions?

The manuscript must describe a technically sound piece of scientific research with data that supports the conclusions. Experiments must have been conducted rigorously, with appropriate controls, replication, and sample sizes. The conclusions must be drawn appropriately based on the data presented. 

Reviewer #2: Yes

**********

3. Has the statistical analysis been performed appropriately and rigorously? 

Reviewer #2: Yes

**********

4. Have the authors made all data underlying the findings in their manuscript fully available?

The PLOS Data policy requires authors to make all data underlying the findings described in their manuscript fully available without restriction, with rare exception (please refer to the Data Availability Statement in the manuscript PDF file). The data should be provided as part of the manuscript or its supporting information, or deposited to a public repository. For example, in addition to summary statistics, the data points behind means, medians and variance measures should be available. If there are restrictions on publicly sharing data—e.g. participant privacy or use of data from a third party—those must be specified.

Reviewer #2: Yes

**********

5. Is the manuscript presented in an intelligible fashion and written in standard English?

PLOS ONE does not copyedit accepted manuscripts, so the language in submitted articles must be clear, correct, and unambiguous. Any typographical or grammatical errors should be corrected at revision, so please note any specific errors here.

Reviewer #2: Yes

**********

6. Review Comments to the Author

Please use the space provided to explain your answers to the questions above. You may also include additional comments for the author, including concerns about dual publication, research ethics, or publication ethics. (Please upload your review as an attachment if it exceeds 20,000 characters)

Reviewer #2: The authors responded well to the revision and the revised version is well put and all of the comments are addressed appropriately .

**********

7. PLOS authors have the option to publish the peer review history of their article (what does this mean?). If published, this will include your full peer review and any attached files.

If you choose “no”, your identity will remain anonymous but your review may still be made public.

**Do you want your identity to be public for this peer review?** For information about this choice, including consent withdrawal, please see our Privacy Policy.

Reviewer #2: No

**********

# Editor report: Acceptance letter

**DOI:** 10.1371/journal.pone.0291835.r004

**Authors:** Amirmohammad Khalaji

12 Sep 2023

PONE-D-23-24761R1 

The application of enhanced recovery after surgery (ERAS) in chronic rhinosinusitis patients undergoing endoscopic sinus surgery: a systematic review and meta-analysis  

Dear Dr. Wu:

I'm pleased to inform you that your manuscript has been deemed suitable for publication in PLOS ONE. Congratulations! Your manuscript is now with our production department. 

If your institution or institutions have a press office, please let them know about your upcoming paper now to help maximize its impact. If they'll be preparing press materials, please inform our press team within the next 48 hours. Your manuscript will remain under strict press embargo until 2 pm Eastern Time on the date of publication. For more information please contact onepress@plos.org.

If we can help with anything else, please email us at plosone@plos.org. 

Thank you for submitting your work to PLOS ONE and supporting open access. 

Kind regards, 

PLOS ONE Editorial Office Staff

on behalf of

Dr. Amirmohammad Khalaji 

Academic Editor

PLOS ONE
